# A host-directed adjuvant sensitizes intracellular bacterial persisters to antibiotics

**DOI:** 10.1038/s41564-025-02124-2

**Published:** 2025-10-10

**Authors:** Kuan-Yi Lu, Xiangbo Yang, Matthew J. G. Eldridge, Rongfeng Sun, Rachel T. Giorgio, Benjamin I. Morris, Nikki J. Wagner, Brian Hardy, Matthew Axtman, Sarah E. Rowe, Xiaodong Wang, Vance G. Fowler, Qingyun Liu, Sophie Helaine, Kenneth H. Pearce, Brian P. Conlon

**Affiliations:** 1 Department of Microbiology and Immunology, University of North Carolina at Chapel Hill, Chapel Hill, NC USA; 2 Center for Integrative Chemical Biology and Drug Discovery, Division of Chemical Biology and Medicinal Chemistry, Eshelman School of Pharmacy, University of North Carolina at Chapel Hill, Chapel Hill, NC USA; 3 Department of Microbiology, Harvard Medical School, Boston, MA USA; 4 Department of Genetics, University of North Carolina at Chapel Hill, Chapel Hill, NC USA; 5 Division of Infectious Diseases, Duke University School of Medicine, Durham, NC USA

**Keywords:** Bacterial host response, High-throughput screening, Antibiotics

## Abstract

Intracellular bacterial reservoirs contribute to antibiotic treatment failure by fostering metabolically dormant persister cells that are highly tolerant to killing. However, strategies to effectively target intracellular persister cells remain limited. Here we developed a high-throughput screen to identify compounds that modulate the metabolic activity of intracellular *Staphylococcus aureus*. The identified compound, KL1, increases intracellular bacterial metabolic activity and sensitizes persister populations of *S. aureus* to antibiotics, without causing cytotoxicity or bacterial outgrowth. KL1 also exhibits adjuvant activity against intramacrophage *Salmonella enterica* Typhimurium and *Mycobacterium tuberculosis*, as well as in murine infection models of *S. aureus* and *S*. Typhimurium infection. Transcriptomic analysis and further mechanistic studies reveal that KL1 modulates host immune response genes and suppresses the production of reactive species in host macrophages, alleviating a key inducer of antibiotic tolerance. Our findings highlight the potential to target intracellular persisters by stimulating their metabolism. There are two major problems in the field of antimicrobial chemotherapy–antibiotic resistance and antibiotic tolerance. Antibiotic tolerance has been frequently connected with poor treatment outcomes in the clinic. Unlike antibiotic resistance, which permits bacterial growth in the presence of drugs, antibiotic tolerance allows bacteria to withstand multiple antibiotics for prolonged periods. The extended survival of tolerant bacteria further predisposes them to evolve antibiotic resistance over time, underscoring the critical need to address antibiotic tolerance. Host interactions have been shown to induce persister formation in numerous pathogens, with the production of reactive oxygen and nitrogen species heavily implicated in the collapse of bacterial metabolic activity and entry into an antibiotic-tolerant state. Yet, tools to study or target this process remain limited. Here we developed a high-throughput screen to identify compounds that modulate intracellular *S. aureus* metabolism, leading to the discovery of KL1, a host-directed compound that sensitizes persisters to antibiotic killing.

## Abstract (web summary)

The authors developed a screen to find compounds that modulate intracellular *Staphylococcus aureus* metabolism and discovered KL1, which sensitizes persisters to antibiotics by reversing host-induced tolerance.

## Main

A principal characteristic of tolerant bacteria is their low energy and metabolic activity, rendering a non-growing state that prevents them from being targeted by antibiotics directed at growth-centred processes^1–6^. Antibiotic tolerance can be induced by the infection environment and activities of innate immune cells. The intracellular environment in macrophages has been strongly associated with the induction of metabolic indolence and antibiotic tolerance^7–11^. Importantly, host-produced reactive oxygen and nitrogen species (ROS/RNS) are responsible for inducing antibiotic tolerance in a wide range of bacterial species, including *S. aureus*, *M. tuberculosis*, *Yersinia pseudotuberculosis* and *S. enterica* Typhimurium^7,8,10–16^. Additional intracellular stressors, such as phagosome acidification and nutrient deprivation, also contribute to tolerance^10^. When conditions become favourable, intracellular bacterial persisters can resume growth, leading to relapsing infections. Thus, strategies to target intracellular persisters are essential for achieving eradication.

To address this difficult-to-eradicate intracellular reservoir, we established a high-throughput screening platform to identify potential metabolic potentiators that sensitize bacteria to antibiotics. We focused on *S. aureus*, a versatile facultative intracellular pathogen capable of surviving in various cell types^17–21^ and frequently causing treatment failure and recurrent infections due to antibiotic tolerance, despite appropriate antibiotic use^22,23^. Among the >4,700 drug-like compounds profiled, we identified KL1 as the lead compound that resuscitates intracellular *S. aureus* and enhances antibiotic efficacy across multiple methicillin-resistant and methicillin-sensitive clinical isolates in mouse and human macrophages, as well as human primary neutrophils. KL1 alone does not promote bacterial outgrowth or show detectable cytotoxicity. It also exhibits adjuvant activity in murine models of *S. aureus* bacteraemia and *S*. Typhimurium infection, as well as intracellular infections of *S*. Typhimurium and *M. tuberculosis*. Using transcriptomic, chemical and biochemical approaches, we discover that rather than directly targeting bacteria, KL1 consistently reduces host ROS/RNS. Together, these findings highlight the potential to pharmacologically sensitize intracellular bacteria by altering the host environment. Our screening platform provides a pathway to uncover host-directed therapies that synergize with antibiotics to clear intracellular persisters.

## Results

### The intracellular environment is an important niche driving antibiotic-tolerant *S*. *aureus*

**Fig. 1:**
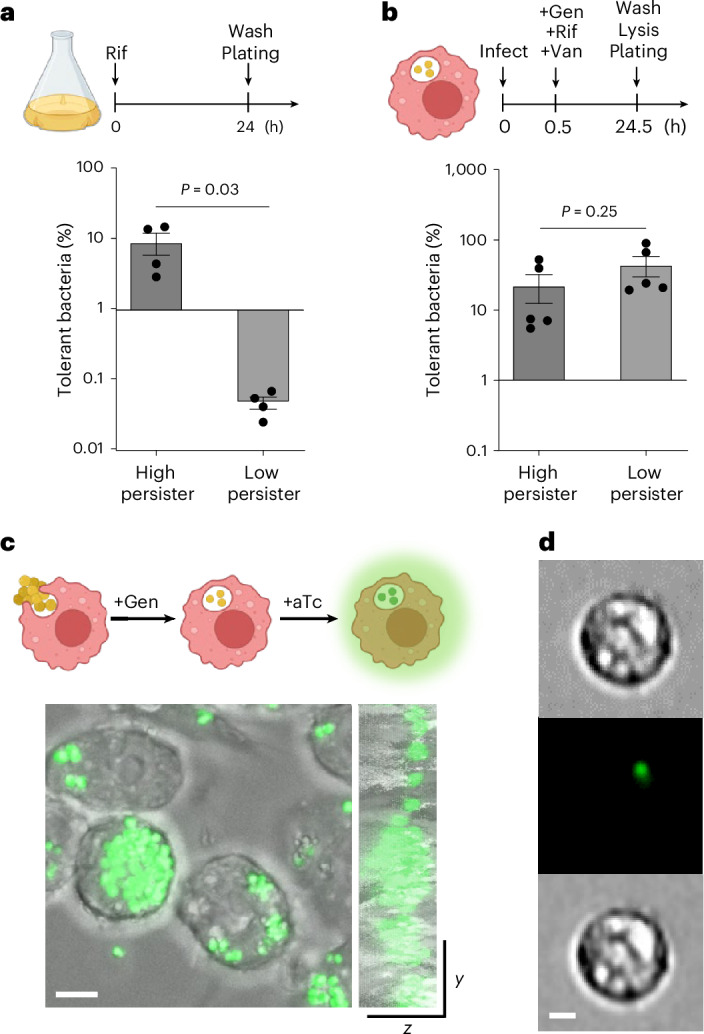
Intracellular environments provide a niche for antibiotic-tolerant *S. aureus* persisters. **a**, Clinical isolates from *S. aureus* bacteraemia patients exhibited variable antibiotic tolerability in planktonic cultures. Rifampicin (Rif) was washed away to enumerate surviving bacteria (c.f.u.s). **b**, High- and low-persister isolates from (**a**) became more tolerant and produced similar numbers of persisters in bone marrow-derived macrophages. Gentamicin (Gen) and vancomycin (Van) were added to eliminate extracellular bacteria. Surviving intracellular bacteria were normalized to c.f.u. counts at the time of antibiotic addition to calculate persister frequencies (*n* = 4, two-sided unpaired *t*-test). Bars represent mean ± s.e.m. Assay schematics are shown above plots. **c**, Confocal *z*-sectioning visualized viable intracellular *S. aureus*. RAW 264.7 macrophages were infected with an inducible GFP reporter strain and treated with 50 µg ml^−1^ Gen to exclude extracellular bacteria. Intracellular bacteria were probed by anhydrotetracycline (aTc) induction. Representative images from 3 independent experiments are shown. Scale bar, 5 μm. **d**, Intracellular persisters in mouse kidneys were detected using ImageStream analysis. C57BL/6J mice were infected with the inducible reporter strain via the intravenous route and intraperitoneally treated with 10 mg kg^−1^ Rif at 1 dpi for 24 h. Kidney cells were extracted at 2 dpi and incubated with 2 µM aTc to induce GFP expression. Representative images from 3 independent experiments are shown. Scale bar, 10 μm. Part of the figure was created with BioRender.com. Source data

Antibiotic tolerance measured in test tubes is often poorly correlated with clinical outcomes, highlighting the importance of probing bacterial drug responses within the context of host–pathogen interactions^24^. Evidence suggests that the host intracellular environment antagonizes antimicrobial agents for obligate and facultative intracellular pathogens, including *S. aureus*^7,9,25–27^. This can be illustrated by testing clinical isolates with varying persister frequencies in tube assays versus cell-based models. We found that although two *S. aureus* isolates displayed a dramatic 200-fold difference in persister formation in tubes, both produced similar high-level antibiotic tolerance after internalization by bone marrow-derived macrophages (BMDMs) (Fig. 1a,b). Both isolates yielded more tolerant bacteria inside macrophages (26–51%) compared with their planktonic cultures (0.05–9%) (Fig. 1a,b). These isolates shared identical minimum inhibitory concentrations (MICs) for rifampicin (6–8 ng ml^−1^) and other antibiotics (Supplementary Table 1). These data indicate that host interaction plays a dominant role in promoting antibiotic tolerance.

As a versatile pathogen, *S. aureus* can adapt to multiple intracellular niches and colonize diverse mammalian cells. To track intracellular persister cells in vivo, we constructed inducible *S. aureus* fluorescent reporter strains and demonstrated the feasibility of using these strains to probe intracellular bacteria. Viable bacteria inside macrophages were visualized via confocal microscopy following induction with anhydrotetracycline (aTc) (Fig. 1c and Supplementary Videos 1–3). To determine whether *S. aureus* survives antibiotic treatment inside mammalian cells, C57BL/6J mice were infected via tail vein (i.v.) with the reporter strains and treated with 10 mg kg^−1^ rifampicin intraperitoneally (i.p.) at 1 day post infection (dpi). Rifampicin was chosen for its ability to penetrate mammalian cells^7^. Kidney cells were extracted at 2 dpi, followed by aTc induction of GFP expression in the presence of gentamicin to exclude extracellular bacteria. Using ImageStream analysis, we observed live cells harbouring GFP-expressing *S. aureus* that survived rifampicin (Fig. 1d). Thus, the intracellular environment not only provides a physical barrier but also promotes antibiotic tolerance. These findings underscore the need to devise strategies to eradicate intracellular persisters, especially given the limited antibiotic options available for intracellular infections.

### A high-throughput screen identifies a compound that sensitizes intracellular *S. aureus* to antibiotics

**Fig. 2:**
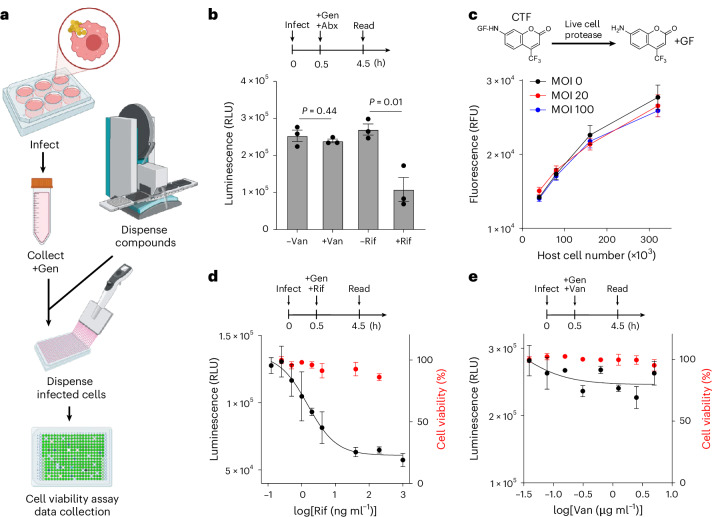
High-throughput screen identifies modulators of intracellular *S. aureus* metabolism. **a**, Schematic of the compound screening platform (created with BioRender.com). **b**, The metabolic activity of intracellular *S. aureus* was monitored using a bioluminescent MRSA strain JE2-lux. RAW 264.7 macrophages were infected with JE2-lux and treated with 20 µg ml^−1^ vancomycin or 10 µg ml^−1^ rifampicin for 4 h before luminescence detection. Gentamicin (50 µg ml^−1^) was included to eliminate extracellular bacteria. Only Rif penetrates host membranes to target intracellular bacteria. Representative of 2 independent experiments (*n* = 3, two-sided unpaired *t*-test). Bars represent mean ± s.e.m. **c**, Host cell viability was assessed using CellTiter-Fluor (CTF), which correlated with viable RAW 264.7 cell numbers (Pearson *r* > 0.97, two-sided *P* = 0.02) and was not affected by *S. aureus*. The MOIs of 0 (black), 20 (red) and 100 (blue) are shown (*n* = 3). Bars represent mean ± s.e.m. **d**,**e**, Dose–response curves for Rif (**d**) and Van (**e**) inhibition of intracellular *S. aureus* activity (black) and corresponding host cell viability (red). Representative of 2 independent experiments (*n* = 3). Bars represent mean ± s.e.m. Assay schematics are shown above plots. Source data

Professional phagocytes, including macrophages, facilitate bacterial clearance during bloodstream infections. However, *S. aureus* can survive this hostile environment after engulfment by adopting a less metabolically active lifestyle^17^. This metabolic indolence favours antibiotic tolerance and persister formation, as most antibiotics target growth-centred processes. To address this, we developed a high-throughput platform to screen compounds that resuscitate intracellular *S. aureus*, aiming to sensitize the intracellular population to antibiotics (Fig. 2a). We used a bioluminescent methicillin-resistant *S. aureus* (MRSA) strain JE2-lux to probe intracellular bacterial metabolic activity^28^. Lux-based bioluminescent reporters are effective for real-time probing of bacterial energy status^29–31^. The lux reaction requires reducing co-factors (NAD(P)H and FMNH_2_), oxygen, and ATP, thus tightly coupling it to cellular metabolism^32^.

**Extended Data Fig. 1:**
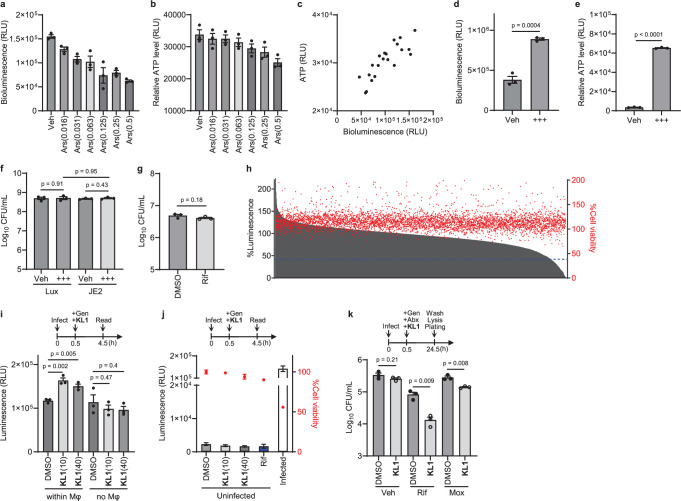
Kinase-targeted screen identifies modulators of intracellular *S. aureus* metabolic activity. The bioluminescent MRSA strain JE2-lux serves as a proxy for bacterial metabolic activity. **a**‒**c**, ATP depletion with sodium arsenate (Ars; 0‒0.5 mM, 30 min) shows a positive correlation between ATP levels and luminescence (Pearson’s *r* = 0.84; two-sided p < 0.0001). ATP was measured in wild-type JE2 using BacTiter-Glo. No change in CFU was observed. Representative of two experiments (n = 3). **d**‒**f**, Nutrient supplementation (1% glucose, 5 mM sodium pyruvate, 0.5% casamino acids (+++) for 30 min) increased bioluminescence in JE2-lux and ATP levels in wild-type JE2, without affecting CFU after nutrient supplementation or between strains. Representative of two experiments (n = 3, two-sided unpaired t-test). **g**, Short-term rifampicin (Rif, 4 h) did not alter intracellular CFU. Representative of two experiments (n = 3, two-sided unpaired t-test). **h**, A library of >4,700 drug-like compounds (10 µM), structurally similar to kinase inhibitors, was screened for intracellular MRSA activity. Luminescence (black bars) was normalized to vehicle (0.1% DMSO). Host cell viability (red circles) was measured by CellTiter-Fluor. Rif-treated cells are shown as reference (blue line). **i**, Infected macrophages (Mφ) were treated with 10 or 40 µM **KL1**. Gentamicin (Gen; 50 µg/mL) was added to eliminate extracellular bacteria. Cultures without Mφ but with Gen and input CFU were controls. Representative of three experiments (n = 3, two-sided unpaired t-test). **j**, Uninfected Mφ treated with **KL1** (10 or 40 µM) were assessed for viability (CellTiter-Fluor; red circles). Rif-treated (blue) and infected (white) macrophages served as controls (n = 3, two-sided unpaired t-test). These data indicate that **KL1**’s phenotype is not due to background noise from the compound, the host cells or dead extracellular bacteria. **k**, CFU data of Fig. 3b. Representative of 2–3 experiments (n = 3, two-sided unpaired t-test). Assay schematics are shown above plots. Bars represent mean ± SEM. Source data

To validate the reporter, we treated JE2-lux and wild-type JE2 with 0–0.5 mM sodium arsenate, which induces ATP depletion^1^, and measured bioluminescence and ATP levels. We observed a dose-dependent correlation between the bioluminescence signal and intracellular ATP levels (Extended Data Fig. 1a–c). Similarly, nutrient supplementation elevated both ATP levels and bioluminescent output without affecting bacterial numbers, supporting the use of lux-based bioluminescence as a readout of metabolic activity (Extended Data Fig. 1d–f). We then evaluated the reporter’s utility intracellularly. Rifampicin reduced the bioluminescence of intracellular bacteria, consistent with its ability to penetrate mammalian cell membranes and inhibit bacterial transcription, thereby reducing metabolic activity (Fig. 2b). In contrast, vancomycin, which does not penetrate mammalian cells, had no effect. Importantly, short-term treatment with rifampicin (4 h) did not reduce intracellular bacterial viability (Extended Data Fig. 1g), consistent with the notion that rifampicin first suppresses bacterial metabolism before killing. This observation suggests that the bioluminescence signal in this context more likely reflects bacterial metabolic activity than total burden. While we cannot fully exclude contributions from bacterial number, we primarily used the reporter to infer bacterial metabolic activity and energy state.

Next, we incorporated a cell viability assay to monitor drug cytotoxicity. We verified that the readout reflected live mammalian cell number and was not influenced by bacterial presence (Fig. 2c). We then assessed the assay’s dynamic range by measuring bioluminescence as a function of rifampicin concentrations. The dose-response showed that 2 ng ml^−1^ rifampicin resulted in 50% reduction in bacterial activity without compromising host viability (Fig. 2d). In comparison, vancomycin had no effect across concentrations (Fig. 2e).

To identify compounds that modulate intracellular *S. aureus* metabolism as antibiotic adjuvants, macrophages infected with JE2-lux were collected from gentamicin-containing media to eliminate extracellular bacteria and dispensed into 384-well plates with various compounds. Bioluminescence and host viability were measured after 4 h. We screened >4,700 drug-like compounds that share structural similarity to kinase inhibitors (Extended Data Fig. 1h). Among them, 77 compounds reduced bioluminescence below rifampicin-treated controls. This reduction may reflect lower bacterial burden or reduced metabolism. Among the 32 compounds that most strongly reduced bioluminescence, 31 did not alter persister frequencies, indicating that they probably suppressed bacterial metabolism rather than killing persisters (Supplementary Fig. 1). The one compound that appeared to reduce bacterial burden did so because infected macrophages were washed away after 24-h treatment, rather than through bactericidal activity against intracellular bacteria.

**Fig. 3:**
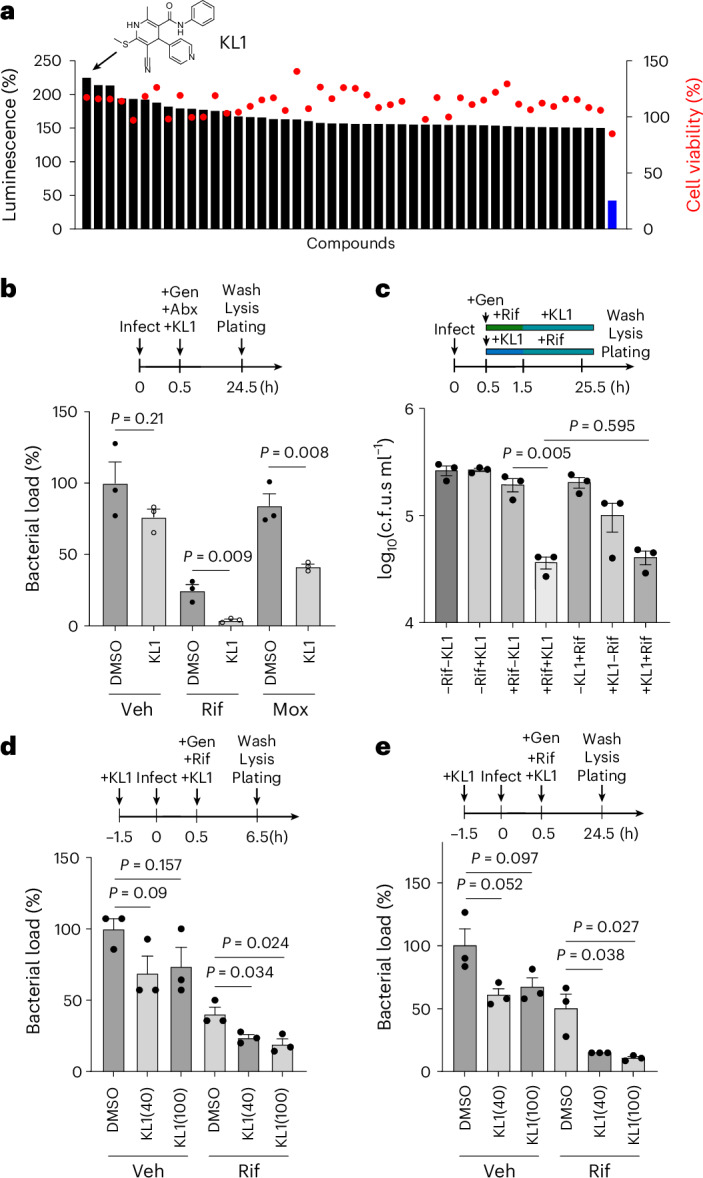
Kinase-targeted screen uncovers intracellular *S. aureus* sensitizer. **a**, Forty-five compounds (10 µM) enhanced luminescence signal by >1.5-fold (black bars) relative to the vehicle control (0.1% DMSO) without causing cytotoxicity (red circles) during a 4-h treatment. The structure of the top candidate, KL1, is shown. Rifampicin-treated cells served as a reference (blue bar). **b**, KL1 (open circles)enhanced the killing activity of Rif (10 µg ml^−1^) and moxifloxacin (Mox; 50 µg ml^−1^) against intracellular MRSA in RAW 264.7 macrophages. Gentamicin (Gen; 50 µg ml^−1^) was included to eliminate extracellular bacteria. Surviving intracellular bacteria were normalized to the untreated control (Gen-only, no antibiotics (Abx), no KL1). Representative data from 2–3 independent experiments (*n* = 3). **c**, KL1’s sensitizing effect was not affected by antibiotic pre-exposure. Infected cells were pretreated with 10 µg ml^−1^ Rif (±Rif±KL1) or 40 µM KL1 (±KL1±Rif) for 1 h, followed by the addition of the complementary treatment. Untreated (−Rif−KL1) served as control. Representative of 3 independent experiments (*n* = 3). **d**,**e**, KL1 retains adjuvant activity in human THP-1-derived macrophages. Cells were pretreated with 0–100 µM KL1, infected at MOI 20, then treated with Rif (10 µg ml^−1^) and 0–100 µM KL1 for 6 h (**d**) or 24 h (**e**). Surviving bacteria were normalized to the Gen-only control. Representative data from 3–4 independent experiments (*n* = 3, two-sided unpaired *t*-test). Bars represent mean ± s.e.m. Assay schematics are shown above plots. Source data

Intriguingly, we identified 45 compounds that increased bioluminescence by >1.5-fold without cytotoxicity (Fig. 3a). The top hit, KL1 (PubChem CID: 2881454), consistently increased the bioluminescence of intracellular *S. aureus* (Extended Data Fig. 1i). This was not due to host cells, gentamicin-killed extracellular bacteria, or KL1 itself (Extended Data Fig. 1i,j). Co-administering KL1 with rifampicin and moxifloxacin enhanced killing of intracellular MRSA by up to 10-fold (Fig. 3b and Extended Data Fig. 1k). Notably, while increasing bacterial metabolism could risk promoting bacterial growth when antibiotic levels fall below inhibitory concentrations, KL1 alone (Veh; no antibiotics) did not induce bacterial outgrowth, supporting its potential as a safe therapeutic adjuvant. Compounds ranked 2–9 at 10 µM did not exhibit adjuvant activity, suggesting that they did not sufficiently enhance metabolic activity to enable sensitization (Supplementary Fig. 2).

**Extended Data Fig. 2:**
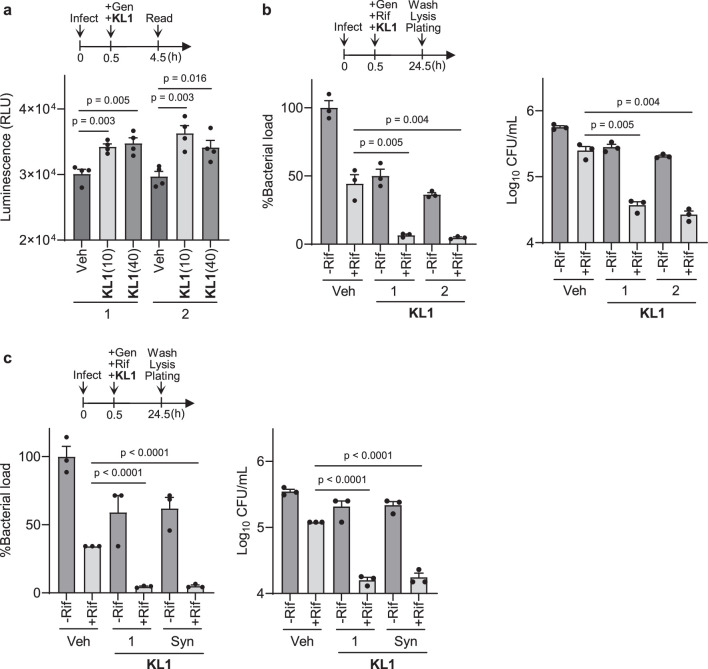
The adjuvant activity of **KL1** was consistent across compound sources. **a**,**b**, **KL1** (10 and 40 µM) from two suppliers, ChemBridge (1) and Enamine (2), consistently elevated the metabolic activity of intracellular *S. aureus* (n = 4) and enhanced rifampicin (Rif; 10 µg/mL) killing (n = 3, two-sided unpaired t-test). **c**, in-house synthesized **KL1** (Syn) showed comparable adjuvant activity to commercial **KL1** (n = 3, two-sided unpaired t-test). Gentamicin (Gen; 50 µg/mL) was used to eliminate extracellular bacteria. CFU counts were normalized to untreated controls (no Rif, no **KL1**, Gen-only). Assay schematics are shown above plots. Bars represent mean ± SEM. Source data

**Extended Data Fig. 3:**
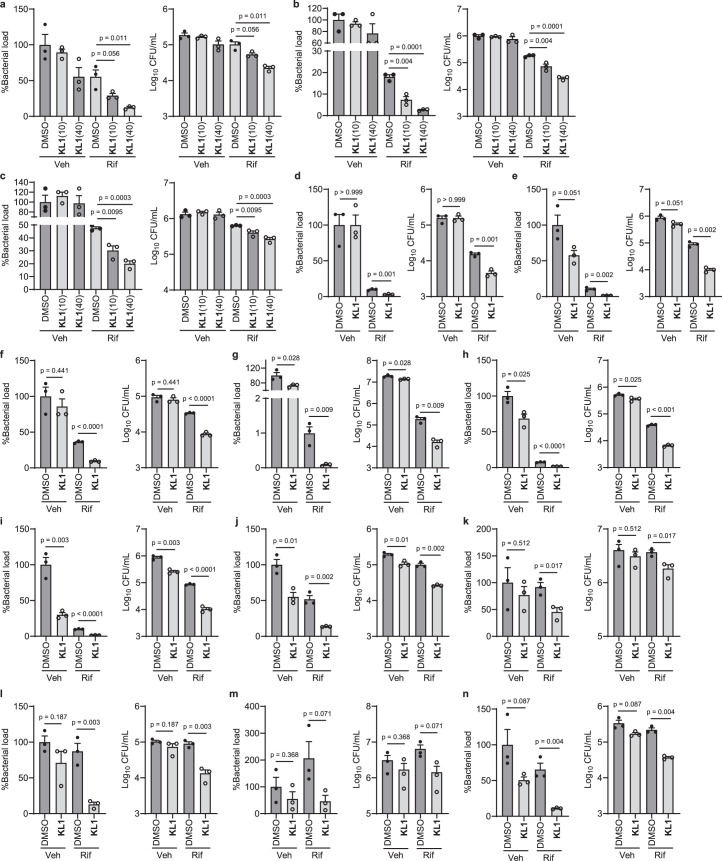
Compound **KL1** sensitizes intracellular MRSA and MSSA to antibiotic killing in a dose-dependent manner. **a**, RAW 264.7 cells were infected with MRSA strain JE2-lux and treated with 10 or 40 µM **KL1** with or without 10 µg/mL rifampicin (Rif). Gentamicin (Gen; 50 µg/mL) was used to eliminate extracellular bacteria. **b,c, KL1**-mediated sensitization was observed in clinical isolates with high (**b**) and low (**c**) persister phenotypes (see Fig. 1a). A dose-dependent response was observed with 0–40 µM **KL1**. Representative data of three experiments (n = 3, two-sided unpaired t-test). **d**–**n, KL1** showed consistent adjuvant activity across multiple MRSA and MSSA isolates: MW2 (**d**), SA03739 (**e**), SA03740 (**f**), SA03758 (**g**), SA03775 (**h**), SA03803 (**i**), SA03809 (**j**), SA03815 (**k**), SA03833 (**l**) and SA03850 (**n**), but not SA03847 (**m**), which was highly Rif-resistant (Supplementary table 1). The number of surviving bacteria was normalized to the untreated control (no Rif, no **KL1**, Gen-only). Corresponding CFU data are shown (right). Representative data of two experiments (n = 3, two-sided unpaired t-test). Bars represent mean ± SEM. Source data

To confirm reliability, we repeated the experiments using KL1 from two independent suppliers and one from in-house synthesis. In all cases, KL1 increased *S. aureus* metabolic activity and rifampicin efficacy (Extended Data Fig. 2). This enhanced killing was observed across lab strains and clinical isolates, and it was proportional to KL1 concentration (Extended Data Fig. 3). KL1 also synergized with rifampicin even when infected macrophages were pretreated with antibiotics, suggesting that low-energy persisters may be resuscitated and sensitized (Fig. 3c).

**Extended Data Fig. 4:**
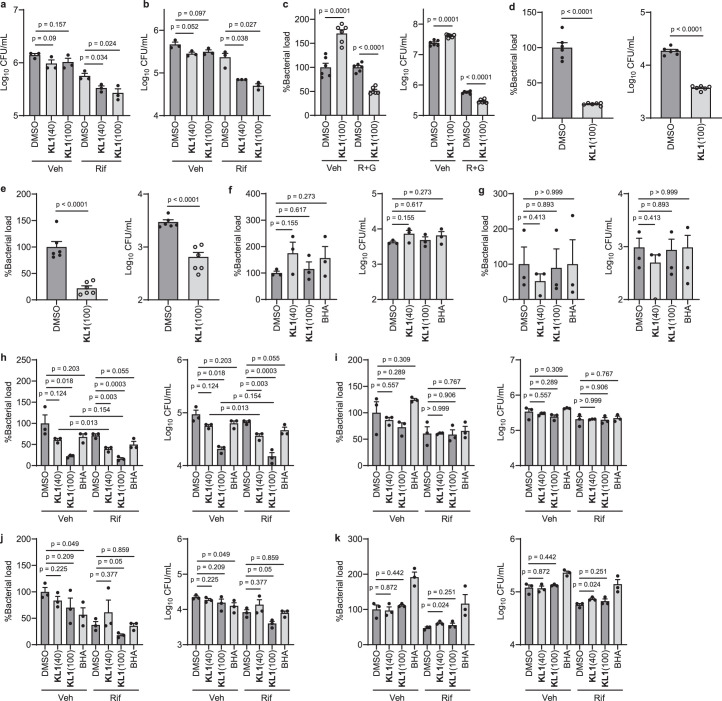
**KL1** enhances antibiotic killing in human primary neutrophils. **a**,**b**, CFU data of Figs. 3d and 3e, respectively. Representative data of 3–4 experiments (n = 3, two-sided unpaired t-test). **c**–**e**, **KL1** increases antibiotic efficacy in human primary neutrophils. Infected neutrophils were treated with 10 µg/mL rifampicin and 25 µg/mL gentamicin (R + G) for 4 h (**c**) or 25 µg/mL moxifloxacin for 13 h (**d**) and 24 h (**e**). Corresponding CFU data shown (right). Representative data of three experiments. Neutrophils were isolated from fresh blood of four healthy donors. **f**–**k**, Human PBMC-derived macrophages do not recapitulate ex vivo ROS/RNS levels, and neither **KL1** (40–100 µM) nor the antioxidant BHA (20 µM; positive control) synergizes with antibiotics in this setting. PBMCs from healthy donors were differentiated with GM-CSF (20–50 ng/mL) for 6 days, followed by polarization with LPS (100 ng/mL) and IFN-γ (20–50 ng/mL) for 1–2 days. Cells with (**g**) or without (**f**) PMA (250–500 nM, 2–3 h) did not phenocopy the adjuvant activity observed with **KL1** and BHA. Representative data from 3–5 experiments (n = 3, two-sided unpaired t-test). **h**–**k**, A shortened differentiation protocol (4-day GM-CSF, 16-h LPS/IFN-γ, 2-h PMA) yielded inconsistent results across blood donors (donor 1: **h**,**j**; donor 2: **i**,**k**) and treatment durations (6-h: **h**,**i**; 18-h: **j**,**k**) (n = 3, two-sided unpaired t-test). Bars represent mean ± SEM. Source data

We next conducted the same experiments using THP-1 cells, a human monocyte-derived macrophage model, and showed that KL1 similarly enhanced antibiotic killing in human macrophages (Fig. 3d,e and Extended Data Fig. 4a,b). In addition, we extended the analysis to human primary neutrophils. Importantly, KL1 sensitized *S. aureus* to both moxifloxacin and rifampicin plus gentamicin killing in neutrophils from healthy donors (Extended Data Fig. 4c–e). Together, we demonstrated the feasibility of pharmacologically sensitizing intracellular *S. aureus* persisters to antibiotics and confirmed that screening these sensitizers using our newly established semi-automated high-throughput platform is viable.

### Compound KL1 exhibits adjuvant activity in a mouse model for *S. aureus* sepsis

**Fig. 4:**
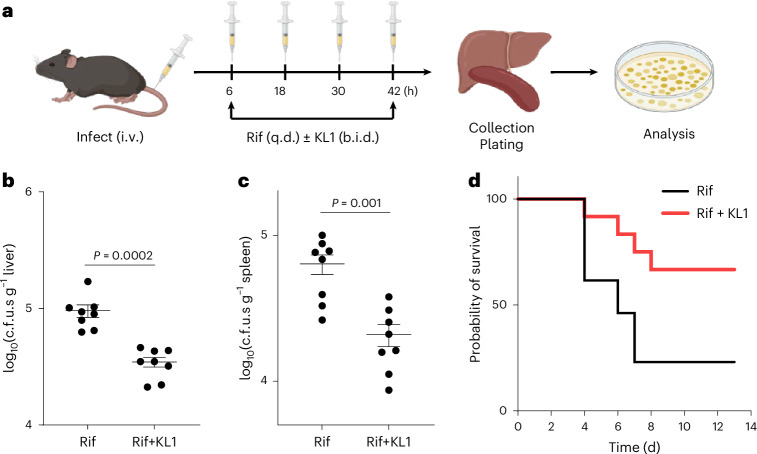
KL1 enhances antibiotic killing of *S. aureus* in a murine bacteraemia model. **a**, Schematic of the murine model. C57BL/6J mice were infected via intravenous (i.v.) injection and treated intraperitoneally (i.p.) with rifampicin (Rif; 10 mg kg^−1^, once a day (q.d.)) ±KL1 (100 mg kg^−1^, twice a day (b.i.d.)) starting at 6 hpi for 2 days. Organs were collected to quantify bacterial burden. Created with BioRender.com. **b**,**c**, Co-administration of KL1 and Rif significantly reduced *S. aureus* c.f.u.s in liver (**b**) and spleen (**c**). Representative of 2 experiments (*n* = 8, Mann–Whitney two-tailed test). **d**, Combined Rif and KL1 improved mouse survival (red) compared to a single dose of Rif alone (black). Each group included 12 (Rif+KL1) or 13 (Rif) mice pooled from 2 independent experiments (*P* = 0.02, Mantel–Cox test). Source data

**Extended Data Fig. 5:**
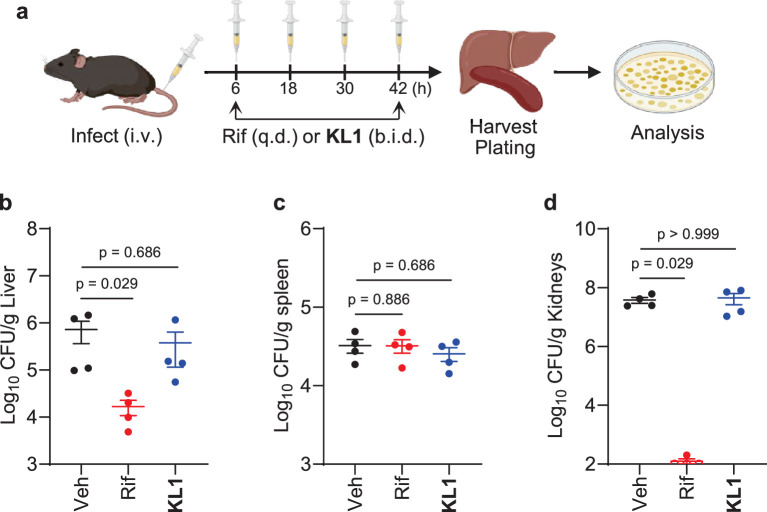
**KL1** alone does not cause bacterial outgrowth in the bacteremia murine model. **a**, C57BL/6 J mice were infected via intravenous (i.v.) tail vein injections and intraperitoneally treated with 10 mg/kg rifampicin (Rif) (q.d.) or 100 mg/kg **KL1** (b.i.d.) at 6 h post-infection (hpi) for 2 days. The vehicle control (Veh) was included for comparison. Created with BioRender.com. **b–d**, The liver (**b**), spleen (**c**) and kidneys (**d**) were collected and homogenized to enumerate the number of surviving bacteria (CFU) (n = 4, Mann–Whitney two-tailed test). Bars represent mean ± SEM. Source data

We next evaluated KL1’s in vivo efficacy using an *S. aureus* bacteraemia model^7^. Briefly, C57BL/6J mice were intravenously injected with *S. aureus* to induce systemic infection. At 6 h post infection (hpi), mice were administered 10 mg kg^−1^ rifampicin once daily, either alone or in combination with 100 mg kg^−1^ KL1 twice daily, via intraperitoneal injections (Fig. 4a). After a 48-h treatment regimen, livers and spleens were collected, and the tissue homogenates were plated to quantify bacterial burden. Our data showed that combined administration of KL1 and rifampicin further reduced the bacterial burden in both organs compared to rifampicin alone (Fig. 4b,c). This experiment was repeated on separate days with male and female mice from different litters, with each group consisting of 6–8 mice, to ensure reproducibility (*n* = 14 per group). Notably, we previously showed that administering Tempol (a potent ROS scavenger) or genetically deleting *Ncf1* (which impairs the respiratory burst) led to bacterial outgrowth in mice^7^. Similarly, macrophage depletion via clodronate liposomes caused *S. aureus* outgrowth in a related model^17^. Here we found that KL1 alone did not induce bacterial outgrowth in the liver, spleen or kidneys, indicating that it did not compromise host immune control over the infection (Extended Data Fig. 5).

To further examine whether KL1 improves treatment outcomes, we established a murine survival assay. Mice were infected with a higher inoculum of MRSA JE2 and treated with rifampicin (1 mg kg^−1^) with or without KL1 (100 mg kg^−1^, two doses). Mice were monitored daily and euthanized upon reaching a humane endpoint or at the conclusion of the study. Interestingly, co-administration of KL1 improved survival rates by 2-fold (*P* < 0.05, *n* > 12 across 2 experiments), suggesting potential therapeutic activity (Fig. 4d).

### KL1 retains adjuvant activity in *Salmonella* and *Mycobacterium* infection models

**Fig. 5:**
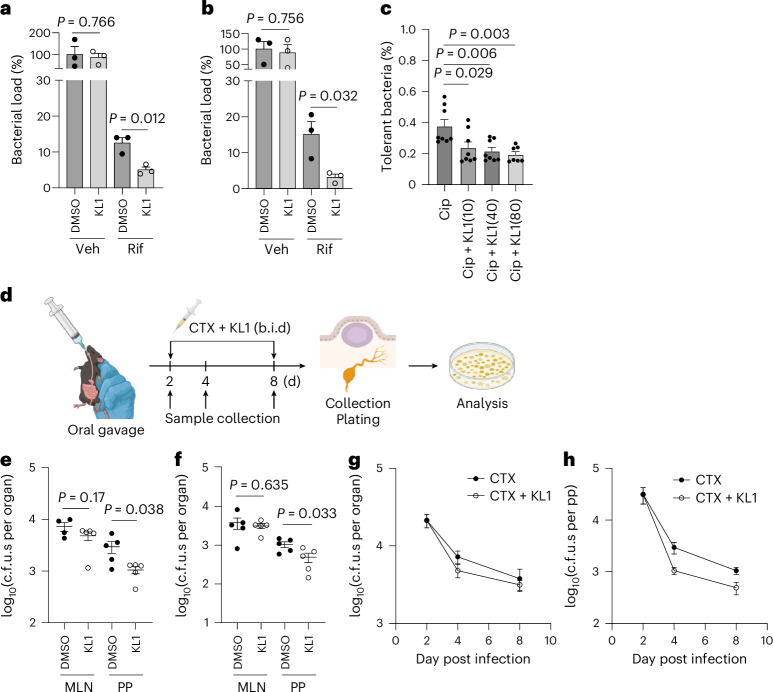
KL1 enhances antibiotic efficacy against *Salmonella* in vivo and *M. tuberculosis* in macrophages. **a**,**b**, KL1 boosted Rif activity against clinical *M. tuberculosis* strains from the globally prevalent lineage 2 (**a**) and lineage 4 (**b**) in macrophages. C.f.u.s were normalized to the untreated control (Gen-only) (*n* = 3, two-sided unpaired *t*-test). **c**, KL1 enhanced ciprofloxacin (Cip; 5 µg ml^−1^) killing of intracellular *S*. Typhimurium. Surviving bacteria were normalized to input c.f.u.s (*n* = 7, two-sided unpaired *t*-test). Bars represent mean ± s.e.m. **d**, Schematic of the *Salmonella* murine infection model. C57BL/6J mice were infected via oral gavage and intraperitoneally treated with 150 mg kg^−1^ cefotaxime (CTX) with or without 100 mg kg^−1^ KL1 (b.i.d.) at 2 dpi for 6 days. Created with BioRender.com. **e**–**h**, Tissue samples were collected to enumerate the number of surviving bacteria (c.f.u.s) at 4 dpi (**e**) and 8 dpi (**f**). Co-administration of KL1 and CTX reduced the bacterial burden in Peyer’s patches (PP) (**e**,**f**,**h**) but not in mesenteric lymph nodes (MLN) (**e**–**g**). A group of 5 mice was examined over the time course (*n* = 5, two-sided unpaired *t*-test). Bars represent mean ± s.e.m. Source data

Since KL1 may or may not directly target the bacteria and may instead modulate host–pathogen interactions, we explored whether KL1 also affects other intracellular pathogens. We opted to investigate *S*. Typhimurium and *M. tuberculosis* because they also survive and form antibiotic-tolerant persister cells within macrophages^10,12,33^. Strikingly, KL1 enhanced antibiotic-mediated killing of both pathogens in macrophages (Fig. 5a–c). In a murine *Salmonella* infection model^34^, we observed a small but significant reduction in bacterial burden in Peyer’s patches at both days 2 and 6 post treatment when antibiotics were combined with KL1 (Fig. 5d–h). These data suggest that KL1 may act as a broad-spectrum antibiotic adjuvant against intracellular bacteria.

### KL1 does not directly act on *S. aureus*

**Extended Data Fig. 6:**
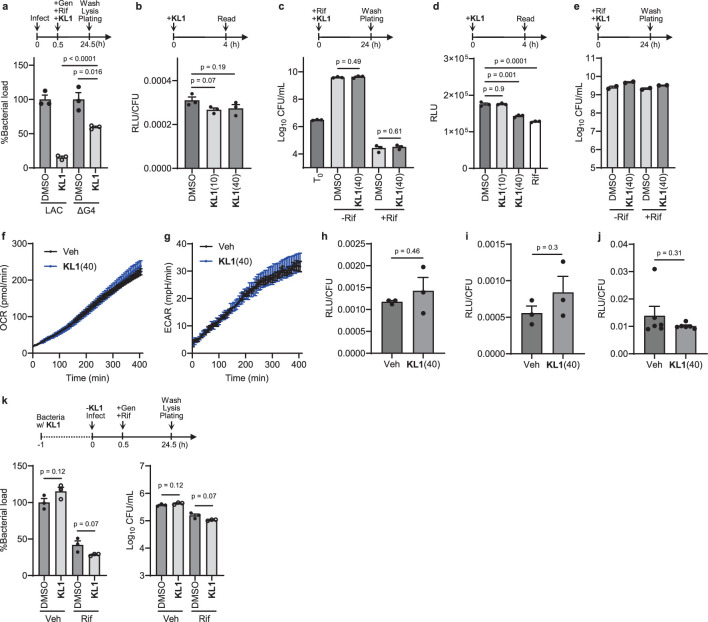
**KL1** does not enhance antibiotic killing or alter metabolism in planktonic bacteria. **a**, Glucose uptake is important for **KL1**-antibiotic synergism. RAW 264.7 cells were infected with wild-type LAC or the ΔG4 mutant lacking four glucose transporters and treated with 40 µM **KL1** and 10 µg/mL rifampicin (Rif). Gentamicin (Gen) was included to eliminate extracellular bacteria. Surviving bacteria were normalized to the control (no **KL1**, Gen- and Rif-only) (black circles). Representative data of two experiments (n = 3, two-sided unpaired t-test). **b**,**d**, Exponential-phase (**b**) and stationary-phase (**d**) *S. aureus* (JE2-lux) cultures were incubated with 10 or 40 µM **KL1** for 4 h, followed by luminescence detection. The vehicle control (0.1% DMSO, black) and rifampicin (Rif; 2 µg/mL)-treated group (blue) served as references. Representative of 2–3 experiments (n = 3, two-sided unpaired t-test). **c**,**e**, Exponential-phase (**c**) and stationary-phase (**e**) cultures were treated with and without 40 µM **KL1** and 2 µg/mL Rif for 24 h, followed by a wash step and plating to enumerate surviving bacteria. Assay schematics are shown above plots. **f**,**g**, **KL1** (40 µM, blue) does not affect oxygen consumption rate (OCR) (**f**) or extracellular acidification rate (ECAR) (**g**) in extracellular *S. aureus*, compared to vehicle control (Veh, 0.1–0.5% DMSO, black). Representative data of four experiments (n = 3). Bars represent mean ± SD. **h**–**j**, Exponential-phase cultures were treated with **KL1** (40 µM) or DMSO (0.1%) for 4 h, followed by measuring relative ATP levels. Luminescent signal was normalized to CFU. *S. aureus* HG003 (**h**), JE2 (**i**) and LAC (**j**) were examined (**h,i**, n = 3; **j**, n = 6, two-sided unpaired t-test). **k**, Pre-treatment of JE2-lux with 40 µM **KL1** before macrophage infection did not enhance antibiotic killing. **KL1** was washed off before infection, indicating host-targeted adjuvant activity. Corresponding CFU data shown (right). Representative of three experiments (n = 3, two-sided unpaired t-test). Source data

Since KL1’s synergy probably depends on metabolism, we tested a mutant MRSA strain lacking all four glucose transporters (ΔG4) to determine whether central metabolism was required^35^. KL1’s adjuvant activity was substantially attenuated in the absence of glucose uptake (Extended Data Fig. 6a), supporting the idea that KL1 enhances antibiotic efficacy by stimulating intracellular bacterial metabolism.

To deconvolute KL1’s mechanism, we interrogated whether KL1 affected extracellular *S. aureus* in planktonic cultures. In actively growing bacteria (mid-exponential phase), KL1 did not consistently increase metabolic activity (Extended Data Fig. 6b). Treatment with KL1 altered neither bacterial growth nor the growth or frequency of persisters after rifampicin treatment (Extended Data Fig. 6c). Likewise, KL1 did not affect metabolic activity or growth of stationary-phase bacteria, with or without antibiotics (Extended Data Fig. 6d,e).

We subsequently employed Seahorse analysis to investigate whether KL1 altered metabolic activity of extracellular *S. aureus*. Our data clearly showed that KL1 had no effect on oxygen consumption rate (OCR) or extracellular acidification rate (ECAR) (Extended Data Fig. 6f,g). In addition, relative ATP levels were unchanged across multiple *S. aureus* strains (Extended Data Fig. 6h–j). Furthermore, pretreatment of *S. aureus* with KL1, followed by washing before macrophage infection, did not enhance antibiotic-mediated killing (Extended Data Fig. 6k). Collectively, our data strongly suggest that KL1 does not act on bacteria but instead perturbs the intracellular environment to alter host–pathogen interaction.

### KL1 modulates host cells and reduces the level of reactive species

**Extended Data Fig. 7:**
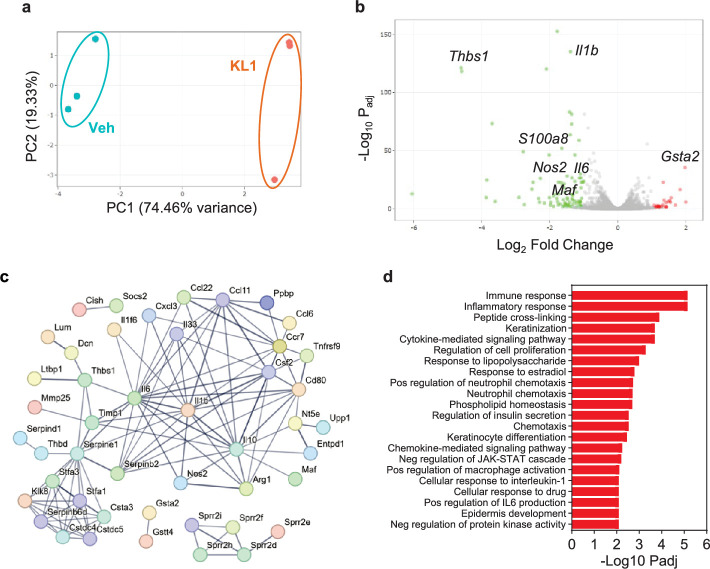
**KL1** alters host immune gene expression linked to reactive species. Principle component analysis (**a**), differential expression (**b**) and String network (**c**) analysis illustrate **KL1**-mediated transcriptional modulation in *S. aureus*-infected macrophages (n = 3). Host genes associated with reactive species production are indicated. Edge thickness represents the confidence in the interactions. Interactions with high confidence (interaction score > 0.7) are shown. **d**, Gene ontology enrichment with an adjusted p-value < 0.01 is shown. Adjusted p-values are calculated using Benjamini–Hochberg false discovery rate method.

To elucidate KL1’s mode of action, we profiled transcriptomic changes in *S. aureus*-infected macrophages following KL1 treatment using bulk RNA-sequencing. Compared with vehicle control, KL1 treatment led to upregulation of 24 host genes and downregulation of 90 host genes (Extended Data Fig. 7). These changes predominantly affected pathways involved in inflammatory responses, cytokine production and macrophage activation. However, KL1 did not appear to obliterate the immune response, and KL1 treatment alone did not cause bacterial outgrowth in either cell-based or murine infection models. Intriguingly, multiple host genes involved in regulating reactive oxygen and nitrogen species (ROS/RNS) were differentially expressed (Extended Data Fig. 7b,c). Key genes promoting ROS/RNS production: *Thbs1*, *S100a8* and *Nos2*, were significantly downregulated, while the glutathione S-transferase gene *Gsta2*, involved in cellular anti-oxidative defence, was upregulated^36,37^. As such, KL1 may alleviate oxidative and nitrosative stress in the intracellular niche, aligning with previous findings that ROS/RNS contribute to antibiotic tolerance and persister formation in *S. aureus* and other bacteria^7,8,10–13,38^.

**Fig. 6:**
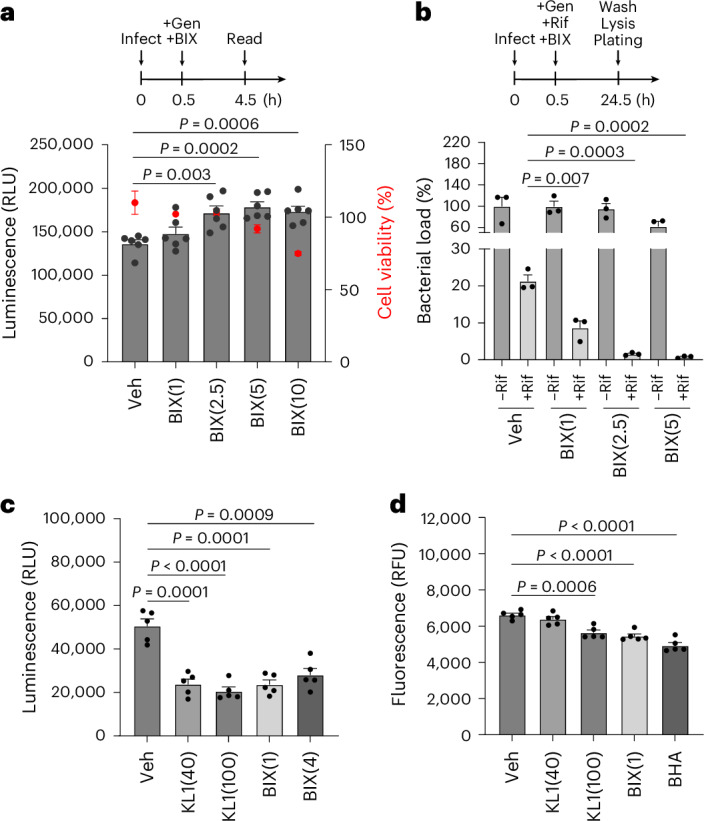
KL1 reduces the production of reactive species upon infection. **a**, Inhibition of EHMT2/G9a phenocopies KL1-mediated sensitization in intracellular *S. aureus*. A selective EHMT2/G9a inhibitor, BIX-01294 (BIX; 1–10 µM), increased the bioluminescence signal (grey bars) of strain JE2-lux compared to the vehicle control (Veh; 0.1% DMSO). Cytotoxicity of BIX was also assessed (red circles). Representative data of 3 experiments (*n* = 6, two-sided unpaired *t*-test). **b**, BIX increased rifampicin (Rif; 10 µg ml^−1^) killing of intracellular MRSA in a dose-dependent manner (1–5 µM). Gentamicin (50 µg ml^−1^) was added to eliminate extracellular bacteria. Surviving bacteria were normalized to the untreated control (no Rif, no BIX, Gen-only). Representative data of 3 experiments (*n* = 3, two-sided unpaired *t*-test). **c**,**d**, Both KL1 (40, 100 µM) and BIX (1, 4 µM) reduced ROS levels at 4 hpi. Chemiluminescent L-012 (**c**) and fluorescent fluorescein-boronate (Fl-B) (**d**) probes were used to quantify reactive species. Antioxidant butylated hydroxyanisole (BHA; 20 µM) served as control. Representative data from 3–4 experiments (*n* = 5, two-sided unpaired *t*-test). Assay schematics are shown above plots. Bars represent mean ± s.e.m. Source data

**Extended Data Fig. 8:**
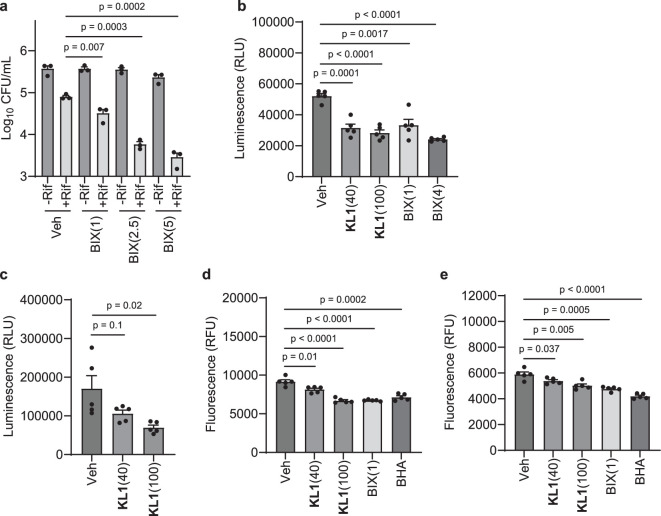
**KL1** reduces reactive species in both infected and uninfected macrophages. **a**, CFU data of Fig. 6b. Representative data of three experiments (n = 3, two-sided unpaired t-test). **b**–**e, KL1** (40, 100 µM) and EHMT2/G9a inhibitor, BIX-01294 (BIX; 1, 4 µM) reduced ROS/RNS levels in *S. aureus*-infected macrophages at 8 hours post-infection (hpi) (**b**,**d**) and in uninfected macrophages (**c**,**e**). L-012 (**b**,**c**) and fluorescein-boronate (Fl-B) (**d**,**e**) were used for detection. DMSO (Veh; 0.25%) and BHA (20 µM) served as controls. Representative data of 3–4 experiments (n = 5, two-sided unpaired t-test). Bars represent mean ± SEM. Source data

Although KL1’s mechanism is not fully characterized, this compound has been tested in hundreds of biological screens (PubChem CID: 2881454)^39^. KL1 showed activity in five assays targeting four proteins (Supplementary Table 2). EHMT2/G9a stood out as a potential target: it is an epigenetic regulator expressed in immune cells, including macrophages, and its previously determined half maximal effective concentration (EC_50_) (PubChem AID: 504332) matches the effect range (10–100 µM) seen in our study^39^. Intriguingly, EHMT2/G9a regulates intracellular innate immunity and macrophage polarization by modulating inflammatory genes such as *Il6* and *Il1b*, and also influences NF-κB and JAK/STAT signalling^40–44^. This downstream regulatory profile resembles the transcriptional shift induced by KL1 (Extended Data Fig. 7). To explore EHMT2/G9a’s role, we examined whether its inhibition would affect the antibiotic susceptibility of intracellular *S. aureus*. Intriguingly, treatment with the selective EHMT2 inhibitor BIX-01294 elevated bacterial metabolic activity and enhanced rifampicin efficacy in a dose-dependent manner, phenocopying KL1’s activity (Fig. 6a,b and Extended Data Fig. 8a).

Given EHMT2/G9a’s role in inflammatory regulation and KL1’s impact on ROS/RNS-related gene expression, we hypothesized that KL1 facilitates antibiotic killing by reducing ROS/RNS production. To test this, we used two detection methods: the chemiluminescent probe L-012 and the fluorescent probe fluorescein-boronate (Fl-B)^8,45^. Both probes consistently showed that KL1 at 40 and 100 µM significantly reduced ROS/RNS levels in *S. aureus*-infected macrophages at 4 and 8 h post treatment (Fig. 6c,d and Extended Data Fig. 8b,d). Notably, BIX-01294 and the antioxidant control butylated hydroxyanisole (BHA) similarly decreased ROS/RNS levels. KL1 also reduced ROS/RNS in uninfected macrophages, highlighting its ability to modulate the host environment (Extended Data Fig. 8c,e).

### Inactive analogue of KL1 loses the ability to modulate reactive species

**Extended Data Fig. 9:**
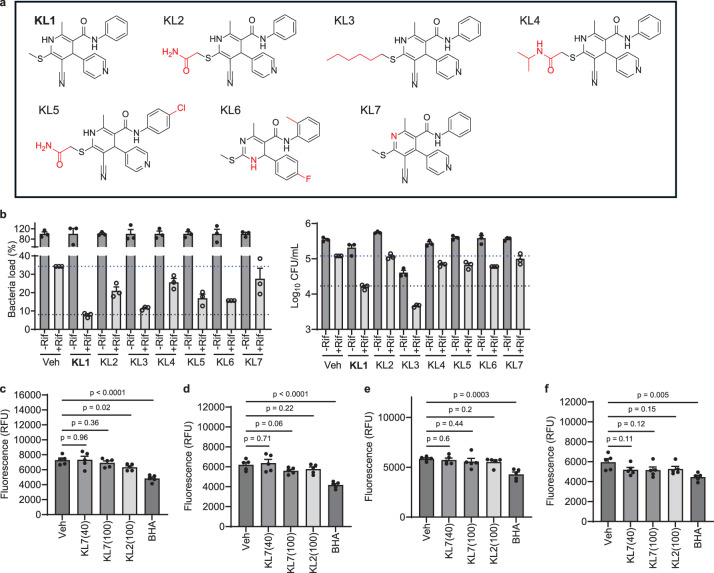
**KL1** analogs with attenuated adjuvant activity lose the ability to regulate reactive species production. **a**, Chemical structures of **KL1** and analogs with modified functional groups highlighted in red. **b, KL1** analogs (40 µM) showed varying abilities to sensitize intracellular *S. aureus* to antibiotic killing. Infected cells were treated with (open circle) and without (solid circle) rifampicin (Rif; 10 µg/mL) and various analogs. Gentamicin (50 µg/mL) was added to eliminate extracellular bacteria. Relative bacterial loads (left) were normalized to the -Rif controls. Representative data of three experiments (n = 3, two-sided unpaired t-test). **c**–**f**, Inactive analog KL7 (40, 100 µM) and weak analog KL2 (100 µM) showed minimal to no effect on ROS/RNS levels in infected (**c**,**d**) and uninfected (**e,f**) macrophages after 4 h (**c**,**e**) and 8 h (**d,f**) treatment. DMSO (Veh) and antioxidant BHA (20 µM) were included as controls. Reactive species were quantified using fluorescein-boronate (Fl-B). Representative of three experiments (n = 5, two-sided unpaired t-test). Bars represent mean ± SEM. Source data

To gain medicinal chemistry insight, we conducted a structure–activity relationship study using six KL1 analogues (Extended Data Fig. 9a). The substitution of carboxamide at the alkyl terminal (KL2, KL4 and KL5) or removal of the nitrile group from the dihydropyridine ring (KL6) moderately reduced their adjuvant activity (Extended Data Fig. 9b). The hexyl substitution on sulfur (KL3) resulted in a high cytotoxicity against host macrophages, indicating a poor therapeutic index. Notably, the 1,4-dihydropyridine ring oxidized analogue (KL7) completely lost the adjuvant activity, presumably due to altered electronic structure, reduced conformational flexibility, or lost hydrogen bonding capacity (Extended Data Fig. 9b). Importantly, KL7 also failed to reduce ROS/RNS in both infected and uninfected macrophages (Extended Data Fig. 9c–f). Altogether, these results strongly support the hypothesis that KL1 enhances intracellular antibiotic efficacy by reducing oxidative and/or nitrosative stress in the host cell.

## Discussion

The ability of bacterial populations or subpopulations of persister cells to survive high concentrations of bactericidal antibiotics for extended periods, despite lacking resistance mutations, is probably a major factor contributing to treatment failure^2,22,24,46–49^. Approximately 20–30% of patients with *S. aureus* bacteraemia fail to clear the infection despite receiving appropriate antibiotic therapy^50–52^. The weak correlation between in vitro antimicrobial susceptibility and clinical outcomes highlights the importance of considering physiological context when addressing poor antibiotic efficacy^24^.

Devising novel strategies to target antibiotic-tolerant persister cells has drawn increasing attention^6,53–57^. Conceptually, eradication of persisters can be achieved either by directly targeting them or by reverting them to a metabolically active state, thereby making them vulnerable to antibiotics. The intracellular environment is a well-established reservoir for persister cells^7–11^. In this study, we demonstrated the potential of modulating the host microenvironment to sensitize intracellular persisters to antibiotics and improve treatment outcomes. Although different stress stimuli can induce antibiotic-tolerant persisters, most produce a metabolically indolent state that can withstand antibiotics. We therefore employed an ATP-dependent bioluminescent reporter to probe metabolic activity of intracellular bacteria as part of a phenotypic screen. When combined with host cell viability and colony-forming unit (c.f.u.) determination, this platform allowed us to identify molecules that stimulate intracellular bacterial metabolism without compromising host viability or expanding the pathogen. Given that the low-energy, low-metabolic activity state is central to antibiotic tolerance in multiple species, this screening may be adapted for other intracellular pathogens across various host cell types and will be agnostic to the mechanism responsible for tolerance^58–61^.

**Extended Data Fig. 10:**
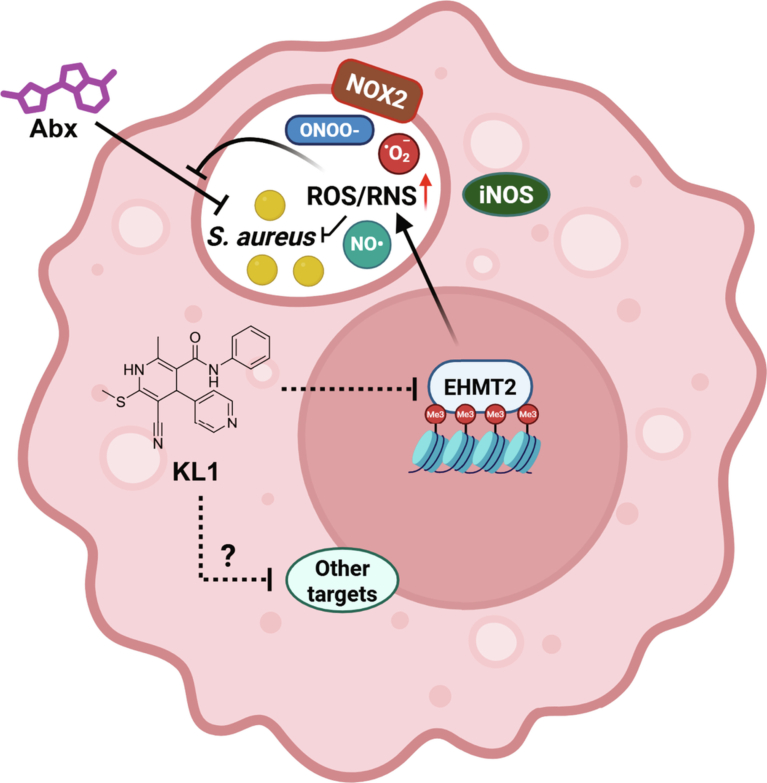
Mechanistic model of **KL1**-mediated sensitization of intracellular *S. aureus.* Host-derived reactive oxygen and nitrogen species (ROS/RNS) help contain intracellular *S. aureus* infections but also antagonize antibiotics by collapsing the bacterial metabolism and forcing the bacteria into antibiotic-tolerant persister cells. The identified compound **KL1** sensitizes intracellular bacteria to antibiotic killing by reducing the production of reactive species upon infection, presumably by targeting the EHMT2/G9a activity. Created with BioRender.com.

Notably, our lead compound KL1 reduced ROS/RNS production in macrophages, sensitized intracellular persisters to antibiotics, and enhanced antibiotic efficacy in murine infection models. This adjuvant activity was observed across three clinically important pathogens in three laboratories. Transcriptomic analysis supports a mechanism involving altered inflammatory responses and reduced ROS/RNS. These observations align with a previous biochemical screen in which KL1 inhibited EHMT2/G9a, which epigenetically regulates diverse processes, including macrophage polarization^40–44^. Inhibiting EHMT2/G9a mirrored KL1’s effect and sensitized intracellular *S. aureus*, suggesting that EHMT2/G9a may be a key mediator of KL1’s effect (Extended Data Fig. 10). Whether KL1 directly binds to and inhibits EHMT2/G9a remains to be determined.

In addition to demonstrating KL1’s activity in THP-1-derived human macrophages and primary human neutrophils, we further examined whether macrophages derived from human peripheral blood mononuclear cells (PBMCs) could recapitulate the response. We tested polarized and non-polarized macrophages, under stimulated and unstimulated conditions, using various differentiation protocols and treatment regimens. However, none produced consistent results (Extended Data Fig. 4f–k). Notably, current evidence strongly supports the notion that human macrophages generate nitric oxide and reactive species in vivo^62^. However, it is notoriously difficult to recapitulate these levels ex vivo, probably due to the prolonged differentiation process or absence of physiological stimuli. This apparent loss of reactive species production ex vivo is further supported by our observation that the antioxidant BHA, used as a positive control, did not enhance antibiotic killing in these conditions (Extended Data Fig. 4f–k).

Together, these findings support that: (1) Targeting host pathways can render intracellular persisters more susceptible to antibiotics; (2) ROS/RNS are key drivers of antibiotic tolerance in vivo; (3) Inhibiting ROS/RNS production can sensitize persisters across bacterial species; and (4) Sensitizing intracellular persisters improves antibiotic efficacy against active infections. This work underscores the potential of host-directed therapeutics, in conjunction with antibiotics, to improve treatment outcomes. Continued development of our screening platform promises identification of additional host-targeted drugs that sensitize intracellular persisters. Ongoing studies on KL1’s mechanism, along with medicinal chemistry optimization and in vivo testing in combination with diverse antibiotics against multiple pathogens, will be critical to determine the full therapeutic potential of this host-directed adjuvant in eradicating deep-seated infections.

## Methods

### Ethics statement

All animal experiments were approved by the UNC Institutional Animal Care and Use Committee (IACUC protocol ID: 24-029.0). Animal housing and feeding were centrally managed by the UNC Division of Comparative Medicine. Human blood was drawn by certified personnel at the UNC Center for AIDS Research and performed in compliance with ethics guidelines set forth by the institution’s Institutional Review Board (IRB). All participants provided informed consent before donating blood.

### General materials

Antibiotics including rifampicin, moxifloxacin hydrochloride, gentamicin sulfate, chloramphenicol (Cam) (>95% purity) were purchased from Fisher Scientific. Vancomycin hydrochloride (>99%) was from Alfa Aesar. Compound KL1 (>90%) was obtained from ChemBridge, Enamine and also synthesized in-house (Supplementary Notes) to validate compound fidelity. Analogues KL2–6 (>90%) were purchased from ChemDiv, ChemBridge and Vitas M Chemical. KL7 was synthesized in-house (>90%) (Supplementary Notes). BIX-01294, an EHMT2/G9a inhibitor (>98%), was from MedChemExpress. RAW 264.7 macrophages (TIB-71) and THP-1 (TIB-202) monocytes were from ATCC and distributed by the UNC Tissue Culture Facility. RAW 264.7 cells were cultured in complete Dulbecco’s modified Eagle medium (DMEM, Gibco) supplemented with 10% (v/v) heat-inactivated fetal bovine serum (FBS) (Avantor Seradigm), 2 mM L-glutamine (Gibco), 1× non-essential amino acids solution (Gibco) and 1 mM sodium pyruvate (Gibco) below 18 passages without exceeding 90% confluency. THP-1 monocytes were maintained in RPMI 1640 medium (Gibco) with 10% FBS, 2 mM L-glutamine and 50 µM 2-mercaptoethanol (Gibco). BMDMs were isolated from C57BL/6J mice (JAX 000664) and cultivated in complete DMEM^63^. Immortalized BMDMs were generated using a CRE-J2 retroviral infection method^64^. Briefly, BMDMs were incubated in 50% (v/v) L929-conditioned medium and infected with CRE-J2 retrovirus on days 5 and 7. Transduced cells were cultured in conditioned medium, gradually reduced to 20%. Immortalized BMDMs were maintained in RPMI 1640 with 2 mM L-glutamine, 1× penicillin–streptomycin (GenClone), 10% (v/v) heat-inactivated FBS (R&D Systems) and 20% conditioned medium.

### Bacterial strains and growth conditions

*S. aureus* strains LAC, HG003, MW2, JE2, JE2-lux (JE2*lux*ABDCE) (kindly provided by Roger Plaut, Center for Biologics Evaluation and Research, Silver Spring)^28^, ΔG4 (kindly provided by Anthony Richardson, University of Pittsburgh, Pittsburgh)^35^ and bacteraemia isolates^8^ were routinely cultured in tryptic soy broth (TSB, Fisher Scientific) or Mueller–Hinton broth (MHB, Oxoid) at 37 °C with shaking (225 r.p.m.) unless otherwise stated. Clinical isolates were obtained under an IRB exemption from a pre-existing collection. *S. enterica* serovar Typhimurium 14028S was cultured in LB broth (Lennox) at 37 °C with shaking. *M. tuberculosis* strains N0155 (lineage 2) and N1283 (lineage 4)^65^ were grown in Middlebrook 7H9 broth (BD Difco) with 10% (v/v) oleic albumin dextrose catalase, 0.2% (v/v) glycerol, 0.05% tyloxapol (w/v) and 0.1 mM sodium propionate (ThermoFisher) to optical density at 600 nm (OD_600_) = 0.6 (ref. ^66^).

### MIC assay

Stationary-phase bacterial cultures were diluted 1:1,000 in MHB containing serially diluted Rif (0–200 µg ml^−1^), Mox (0–20 µg ml^−1^) or Van (0–16 µg ml^−1^) in 96-well assay plates (Corning) in triplicates (200 µl per well). Plates were sealed with Breathe-EASIER membranes (Diversified Biotech) and incubated statically at 37 °C for 24 h. The MICs were determined by the absence of bacterial growth. Three independent assays were performed to ensure reproducibility.

### Construction of the inducible reporter strains

The plasmid pALC2084, which contains a tetracycline (Tet)-inducible GFP-expressing cassette^67^ (kindly provided by Ambrose Cheung, Dartmouth College, Hanover), was first transformed into *S. aureus* strain RN4220 via electroporation using a Gene Pulser Xcell system (Bio-Rad)^68^. The plasmid was then purified and transformed into the recipient *S. aureus* strain HG003. Single colonies were isolated and cultured in TSB containing 10 µg ml^−1^ Cam and 0–2 µM aTc (Sigma) for 2–3 h. GFP induction was verified at excitation and emission wavelengths of 475 nm and 508 nm, respectively, using a Synergy H1 microplate reader (BioTek). For mKate reporter construction, the mKate2 gene was amplified from pRN10 (Addgene, plasmid 84454)^69^ using a specific primer pair (Supplementary Table 3) and Q5 High-Fidelity DNA Polymerase (New England Biolabs). The PCR product was purified using a MinElute kit (Qiagen). Both the Tet-inducible vector pRMC2 (Addgene, plasmid 68940)^70^ and the mKate insert were digested with KpnI-HF and EcoRI-HF (New England Biolabs), gel purified using a QIAquick PCR purification kit (Qiagen), and ligated using T4 DNA ligase (New England Biolabs) at 16 °C for 16 h. The construct was transformed into MAX Efficiency DH5α competent cells (ThermoFisher). Site-directed mutagenesis was performed using a QuikChange kit (Agilent) and a primer pair (Supplementary Table 3) to incorporate an upstream ribosomal binding site. The final pRMC2–mKate plasmid was verified by restriction digest and Sanger sequencing, and then transformed into RN4220 and subsequently HG003. aTc-induced mKate expression was confirmed at 633 nm (excitation 588 nm) using the Synergy H1 reader.

### Live-cell microscopy

RAW 264.7 macrophages were seeded in Nunc 8-well Lab-Tek chambered coverglass (ThermoFisher) at 2–3 × 10^5^ cells per well in 500 µl complete minimum essential medium (MEM, Gibco) supplemented with 10% (v/v) heat-inactivated FBS and 2 mM L-glutamine at 37 °C with 5% CO_2_ for 16 h. Cells were infected with Tet-inducible GFP- or mKate-expressing *S. aureus* at a multiplicity of infection (MOI) of 20 (no Rif) or 100 (with Rif) by centrifugation at 1,000 *g* for 2 min, followed by incubation at 37 °C for 35 min. After washing once in 500 µl complete MEM, cells were treated with or without 10 µg ml^−1^ Rif in the presence of 50 µg ml^−1^ Gen at 37 °C for 1.5 h (no Rif) or 4 h (with Rif). Rif-treated cells were washed three times and incubated in Gen-containing medium (50 µg ml^−1^) at 37 °C for 2–3 h to allow resuscitation of intracellular persisters. Cells were then treated with 2 µM aTc in the presence of 50 µg ml^−1^ Gen at 37 °C for 3 h to induce reporter expression. GFP-expressing *S. aureus*-infected macrophages were imaged using a Leica SP8X Falcon microscope with excitation at 484 nm (WLL2), emission collection at 495–570 nm and a detector gain of 100 V. Imaging parameters included: 1,024 × 1,024-pixel scan, 16-bit depth, speed of 600, pixel averaging of 2, pinhole of 1 AU and acquisition using a ×63 oil-immersion lens (NA 1.4, 1–4× zoom).

Macrophages infected with mKate-expressing *S. aureus* were stained with 100–300 nM LysoTracker Green DND-26 (ThermoFisher) at 37 °C for 30 min, then washed and maintained in 200 µl complete MEM containing 1 µg ml^−1^ Hoechst 33342, 50 µg ml^−1^ Gen and 2 µM aTc. Live-cell *z*-stack and time-lapse imaging were performed using an Olympus FV3000RS confocal microscope (Olympus) equipped with a stage-top incubator at 37 °C, 5% CO_2_ and 100% humidity. The settings were as follows: (1) 405-nm (Hoechst 33342), 488-nm (LysoTracker Green) and 594-nm (mKate) diode lasers were used for excitation with GaAsP detectors; (2) a 516 × 516-pixel scan was applied; (3) pixel averaging was set to 10; (4) pinhole size was set to 1 AU; and (5) images were obtained using a ×60 oil-immersion objective lens (NA 1.4). Images were exported using ImageJ/FIJI (v.1.54)^71^ without gamma correction.

### ImageStream analysis

Cells were loaded into an Amnis ImageStreamX Mark II system (EMD Millipore) for imaging and fluorescence detection. For capturing live cells harbouring viable GFP-expressing *S. aureus*, 405-nm and 488-nm colinear lasers were set to 40 mW and 70–90 mW for live/dead cell discrimination and GFP expression, respectively. In-focus single-cell images were acquired with a ×60 magnification and a low-speed flow rate using the INSPIRE software (EMD Millipore). The exported data were analysed using IDEAS 6.2 software (EMD Millipore).

### High-throughput screen for intracellular *S. aureus* energy modulators

RAW 264.7 macrophages were seeded in 4 ml complete MEM in Costar 6-well tissue culture-treated plates (Corning) at 8 × 10^5^ cells per well and incubated at 37 °C and 5% CO_2_ for 16 h. Cells were infected with *S. aureus* strain JE2-lux at an MOI of 100 for 25 min at 37 °C. Infected cells were washed once with 2 ml PBS and incubated with 10 mM EDTA in PBS (2 ml per well) at 37 °C for 5–10 min to detach. Cells were pelleted at 300 *g* for 6 min, resuspended in complete MEM with 50 µg ml^−1^ Gen at 2.5 × 10^5^ cells per ml, and dispensed (80 µl per well) into 384-well white and black plates (ThermoFisher) for luminescence and fluorescence measurements, respectively. Uninfected macrophages were added to column 1 and 24 as background and viability controls. Plates were preloaded with 80 nl of 10 mM compounds using a Mosquito LV pipetting system (SPT Labtech). Vehicle (0.1% dimethylsulfoxide (DMSO)) and 10 µg ml^−1^ Rif were included for quality control. After cell dispensing, plates were centrifuged at 500 *g* for 2 min and incubated at 37 °C and 5% CO_2_ for 4 h. Luminescence was recorded at 1.25-mm depth with a detector gain of 250 using a Synergy H1 reader. To assess cell viability, 10 µl CellTiter-Fluor (Promega) was added to black plates and incubated for 30 min in the dark before fluorescence was measured at 7-mm depth (gain 100). The kinase-targeted compound library (>4,700 rule-of-five-compliant compounds) was from the UNC Center for Integrative Chemical Biology and Drug Discovery (CICBDD) (Supplementary Table 4). Luminescence and fluorescence signals were normalized to 0.1% DMSO controls. EC_50_ values for Van and Rif against intracellular MRSA strain JE2-lux were determined using GraphPad Prism. The screen was performed once for hit identification.

### Antibiotic survival assays

For *S. aureus* planktonic cultures, 16- to 18-h stationary-phase cultures were diluted 1:100 in MHB and incubated at 37 °C with shaking at 225 r.p.m. for 3 h to reach the mid-exponential phase. Cultures were then treated with 10 µg ml^−1^ Rif and/or 0–40 µM KL1 and incubated for 24 h under the same conditions. Bacteria were washed in one volume of 1% NaCl three times at 20,000 *g* for 5 min, followed by resuspension in one volume of 1% NaCl. Suspensions were serially diluted 10-fold and plated on tryptic soy agar (TSA) to quantify surviving *S. aureus*. Tolerant bacterial frequencies were calculated by normalizing c.f.u.s to the initial input.

To evaluate intracellular *S. aureus* persister frequencies, RAW 264.7 macrophages or BMDMs were seeded into 24-well tissue culture-treated plates (Corning) at 4 × 10^5^ cells per well in 500 µl complete MEM, and incubated at 37 °C and 5% CO_2_ for 16 h. THP-1 cells were seeded at 2 × 10^5^ cells per well in complete RPMI 1640 medium with 100 nM phorbol 12-myristate 13-acetate (PMA) (Cayman) and differentiated into macrophages for 3 days, then rested in PMA-free medium for 16 h before infection. Macrophages were infected with *S. aureus* strains at MOI 10–20 via centrifugation at 1,000 *g* for 2 min, followed by 35 min incubation at 37 °C. After removing the spent medium, cells were incubated in 500 µl complete MEM containing antibiotics (10 µg ml^−1^ Rif, 50 µg ml^−1^ Mox or 20 µg ml^−1^ Van) with or without 0–100 µM KL1 or its analogues (KL2–7), 20 µM BHA (Sigma) or 0–10 µM BIX-01294, in the presence of 50–100 µg ml^−1^ Gen at 37 °C for 6–24 h. Cells were washed three times with 1 ml PBS and lysed with 200 µl 0.5% (v/v) Triton X-100 (Fisher Scientific) at 37 °C for 5 min, followed by the addition of 800 µl PBS. Released intracellular bacteria were serially diluted and plated on TSA. Persister frequencies were normalized to the intracellular c.f.u.s at the time of antibiotic exposure. For comparisons, final bacterial loads were normalized to untreated controls.

For *Salmonella* infections, immortalized BMDMs were seeded in 12-well plates (4 × 10^5^ cells per well) in RPMI 1640 supplemented with 2 mM L-glutamine and 10% FBS at 37 °C and 5% CO_2_ for 24 h. Stationary-phase *Salmonella* Typhimurium were opsonized with 8% (v/v) mouse serum in RPMI 1640 for 20 min and used to infect immortalized BMDMs at MOI 15 (ref. ^26^). Plates were centrifuged at 110 *g* for 5 min, then incubated at 37 °C for 30 min. Cells were washed once with PBS and treated with 5 µg ml^−1^ ciprofloxacin (MP Biomedicals) in the presence of 10–80 µM KL1 or 0.2% DMSO for 24 h. Cells were washed three times in 500 µl PBS, lysed with 0.2% Triton X-100 at 37 °C for 10 min and plated on LB agar. Persister frequencies were calculated as c.f.u.s at 24 h (*T*_24_) over input c.f.u.s (*T*_0_).

For *M. tuberculosis*, bacterial cultures were washed twice with PBS, passed through a 5-µm filter and resuspended in DMEM^72^. BMDMs were infected at MOI 1 for 4 h, washed three times with PBS and treated with 200 µg ml^−1^ Gen for 2 h. Cells were then incubated in DMEM containing 0.1% DMSO, 1 µg ml^−1^ Rif, 100 µM KL1, or a combination of Rif and KL1 in the presence of 20 µg ml^−1^ Gen in triplicate at 37 °C for 3 days. Cells were washed three times with PBS, then lysed with 200 µl of 0.1% Triton X-100 for 5 min. The extracted intracellular bacteria were plated on Middlebrook 7H10 agar plates (BD Difco). The number of viable bacteria was enumerated after 21 days of incubation at 37 °C and 5% CO_2_.

### Assessment of adjuvant activity in primary human neutrophils

Blood samples from healthy donors were processed immediately following a standard protocol^73^. Whole blood was mixed 1:1 with 3% dextran T-500 (Pharmacosmos)/0.9% NaCl and incubated at room temperature for 20 min to sediment erythrocytes. Leucocyte-rich supernatants were transferred to 50-ml tubes and centrifuged at 250 *g* for 10 min at 4 °C. Pellets were resuspended in 0.9% NaCl equal to the starting blood volume. Ficoll-Paque Plus (Cytiva, Fisher Scientific) was underlaid (10 ml per tube) beneath the cell suspension, followed by centrifugation at 400 *g* for 40 min at 20 °C (no brake). Pellets were resuspended in 20 ml cold 0.2% NaCl for 30 s, followed by addition of 20 ml cold 1.6% NaCl to lyse remaining erythrocytes. Neutrophils were pelleted at 250 *g* for 6 min, washed in RPMI 1640 with 2% FBS, 2 mM L-glutamine and 10 mM HEPES, and centrifuged at 250 *g* for 4 min. Cells were resuspended in the same medium, rested at 37 °C for 0.5–1 h, filtered through 100-μm strainers (VWR) and dispensed at 1 × 10^5^ neutrophils per well (500 µl) in 24-well ultra-low attachment plates. Cells were pretreated with 100 µM KL1 or 0.25% DMSO at 37 °C for 1–1.5 h in 6 replicates, infected with *S. aureus* JE2-lux (MOI 10) at 37 °C for 1 h, followed by treatment with 50 µg ml^−1^ Mox or 10 µg ml^−1^ Rif and 25 µg ml^−1^ Gen for 4 h. Cell suspensions were serially diluted and plated on TSA with 0.4% activated charcoal (Sigma) to neutralize residual antibiotics, allowing enumeration of surviving bacteria. Neutrophils from ≥4 donors were tested.

### Antibiotic survival assays in human PBMC-derived macrophages

Human PBMCs were isolated from fresh blood samples using Ficoll-Paque density gradient centrifugation, as described for neutrophil isolation. The interphase was collected into five volumes of complete RPMI 1640 and centrifuged at 400 *g* for 10 min. Cells were washed twice with 20 ml PBS at 300 *g* for 5 min and rested in complete medium at 37 °C for 30 min. Debris was removed using 100-μm cell strainers. For differentiation, 1–2 × 10^5^ PBMCs were seeded in 1 ml complete medium containing 1× penicillin–streptomycin and 20–50 ng ml^−1^ GM-CSF (PeproTech) per well in 24-well plates for 4–6 days, with medium changed every other day. On day 4 or 6, macrophages were polarized with or without 100 ng ml^−1^ lipopolysaccharide (LPS) (Sigma) and 20–50 ng ml^−1^ IFNγ (PeproTech) for 1–2 days. Polarized macrophages were stimulated with 500 nM PMA at 37 °C for 2–3 h and washed three times in RPMI 1640. Macrophages (polarized and non-polarized, stimulated and unstimulated) were pretreated with 40–100 µM KL1, 20 µM BHA or 0.1–0.25% DMSO in 500 µl complete medium at 37 °C for 1 h in triplicates. Macrophages were infected with *S. aureus* at MOI 10 by centrifugation at 1,000 *g* for 2 min and incubated at 37 °C for 35 min. Cells were subsequently treated with 10 µg ml^−1^ Rif and 50–100 µg ml^−1^ Gen in the presence of KL1, BHA or DMSO, and incubated at 37 °C with 5% CO_2_ for 6–18 h. Cells were washed three times with PBS, lysed in 200 µl 0.5% Triton X-100 for 5 min, diluted with 800 µl PBS and plated on TSA.

### *S. aureus* murine infection and isolation of kidney cells

To visualize viable intracellular *S. aureus*, C57BL/6J female mice (6–9 weeks old) were systemically infected with Tet-inducible GFP-expressing *S. aureus* strain HG003 (5 × 10^6^ c.f.u.) via tail vein injection^7^. At 1 dpi, mice received 10 mg kg^−1^ Rif in 2.5% DMSO, 12.5% PEG300 (Sigma) and 85% sterile H_2_O via intraperitoneal injection. Control mice received vehicle (2.5% DMSO/12.5% PEG300) only. At 2 dpi (24-h Rif treatment), kidneys were homogenized in 5 ml cold PBS with DNase I (50 U ml^−1^, bovine pancreas; ThermoFisher) using a Stomacher 80 Biomaster (Seward) at fast speed for 2 min twice^74^. Homogenates were filtered through 70-µm strainers and pelleted at 300 *g* for 5 min at 4 °C. After three PBS washes, cells were resuspended in cold PBS with 1% FBS and 50 µg ml^−1^ Gen, with or without 2 µM aTc, for 3 h. Samples were stained with LIVE/DEAD fixable violet dead cell stain (1 µl ml^−1^; ThermoFisher) at 4 °C for 30 min before ImageStream analysis.

To evaluate KL1 adjuvant activity in vivo, mice (male:female = 1:1) were infected with 2 × 10^7^ c.f.u.s of strain HG003 via intravenous injection. At 6 hpi, mice were intraperitoneally treated with 10 mg kg^−1^ Rif, alone or with 100 mg kg^−1^ KL1 in 7% DMSO, 40% PEG300 and 53% sterile H_2_O. Rif and KL1 were given once daily and every 12 h, respectively, for 2 days. Organs were homogenized either by repetitively rolling a serological pipette over the samples in sample bags (liver) or by bead beating using a Precellys 24 Touch homogenizer (Bertin Technologies) at 5,000 r.p.m. for 25 s twice with a 5-s interval (spleen and kidney). Homogenates were serially diluted in 1% NaCl and plated on TSA to enumerate surviving bacteria. Bacterial burden (c.f.u.s g^−1^) was normalized to tissue weight. Two independent experiments using 6–8 mice each (14 mice per group in total) from different litters were conducted.

For Kaplan–Meier survival analysis, mice (female) were infected with 2 × 10^8^ c.f.u.s of *S. aureus* JE2-lux. At 6 hpi, mice received either 100 mg kg^−1^ KL1 or the vehicle (7% DMSO, 40% PEG300 and 53% sterile H_2_O). At 12 hpi, a single dose of 1 mg kg^−1^ Rif, alone or with 100 mg kg^−1^ KL1, was administered. Mice were monitored twice daily for signs of disease and euthanized when they reached humane endpoints, defined as change in mobility, severe lethargy, dehydration and a loss of >20% body weight. Survival was recorded until natural death or humane euthanasia. Data from 2 independent experiments (12–13 mice per group) were pooled. Significance was determined using Mantel–Cox test.

### *Salmonella* Typhimurium murine infection

*S*. Typhimurium cultures were back diluted to OD_600_ = 0.1 in LB broth and grown to exponential phase at 37 °C with shaking. Bacterial cells were washed three times with PBS before infection. Female C57BL/6J mice (10 weeks old) were fasted for ≥4 h and infected orally with 1 × 10^10^ c.f.u.s in 200 µl PBS. At 2 dpi, mice received 150 mg kg^−1^ cefotaxime (CTX), alone or with 100 mg kg^−1^ KL1 in 7% DMSO, 40% PEG300 and 53% sterile H_2_O via intraperitoneal injection, given twice daily for 2 and 6 days. Organs were homogenized in PBS using a Mixer Mill MM400 (Retsch) with 3.2-mm stainless steel beads (BioSpec) at 30 Hz for 2 min. Homogenates were serially diluted and plated on LB agar to enumerate surviving bacteria. Five mice per group were used.

### Reactive species assays

Reactive species were measured using the luminescent probe L-012 (Wako Chemical) and the fluorescent dye fluorescein-boronate (Fl-B)^63^. For L-012 probing, RAW 264.7 macrophages were seeded in 100 µl complete MEM in Falcon 96-well white plates (Corning) at 3.84 × 10^4^ cells per well and incubated at 37 °C for 16 h. Cells were infected with *S. aureus* JE2 at MOI 10 by centrifugation at 1,000 *g* for 2 min, followed by a 40-min incubation at 37 °C. Cells were treated with 40–100 µM KL1 or 1–10 µM BIX-01294 in the presence of 50 µg ml^−1^ Gen in 5 replicates for 4–8 h. After three washes with 200 µl prewarmed PBS, 100 µl of 300 µM prewarmed Hanks’ balanced salt solution (Gibco) was added, and luminescence was immediately measured at 37 °C using a Synergy H1 reader. Two consecutive reads were averaged.

For Fl-B staining, macrophages were similarly seeded in 96-well black clear-bottom plates. After treatment with 40–100 µM KL1, 100 µM KL2, 40–100 µM inactive analogue KL7, 1–10 µM BIX-01294 or 20 µM BHA, infected cells were washed twice with 200 µl PBS and incubated with 100 µl of 25–50 µM Fl-B in PBS at 37 °C for 30 min. Following three washes, fluorescence was measured at 535 nm emission with 485 nm excitation (gain 100, area scan) using the Synergy H1 reader.

### ATP measurement

*S. aureus* strains LAC, HG003 and JE2 were grown in TSB for 16–18 h and subcultured at 1:1,000 to 1:40,000 dilution in fresh TSB containing 40 µM KL1 or 0.1–0.5% DMSO in 6 replicates at 37 °C for 4 h. Cultures (100 µl per well) were aliquoted into Falcon 96-well white plates and mixed 1:1 with BacTiter-Glo reagent (Promega), then incubated in the dark on a rocker at room temperature for 5–20 min. Luminescence was measured at 1.25-mm depth with gain 250 using a Synergy H1 reader and normalized to c.f.u.s for relative ATP levels.

To correlate lux-based bioluminescence and bacterial metabolic activity, stationary-phase JE2 and JE2-lux cultures were subcultured at 1:200 in MHB, incubated at 37 °C for 3 h with shaking, then treated with 0–0.5 mM sodium arsenate (Sigma) for 30 min. Parallel cultures diluted 1:6 in PBS were incubated at 37 °C with shaking for 3 h, then treated with 1% glucose, 5 mM sodium pyruvate and 0.5% casamino acids, or vehicle for 30 min. Bioluminescence (JE2-lux) and ATP levels (JE2) were measured immediately. Bacterial suspensions were plated for c.f.u. enumeration.

### Seahorse analysis

This analysis measures real-time changes in cellular metabolism and has been applied to study *S. aureus* respiration^75^. Stationary-phase cultures were diluted 1:100 in fresh TSB in triplicate and incubated for 2–3 h until OD_600_ reached 0.3. Cultures were then diluted 1:500 to 1:1,000 in TSB or Seahorse XF DMEM (Agilent) supplemented with 100 mM glucose, 2 mM L-glutamine and 1 mM sodium pyruvate, and dispensed into poly-D-lysine (PDL)-coated Seahorse XF HS miniplates (100 µl per well). PDL coating involved adding 100 µg ml^−1^ PDL in sterile H_2_O for 30 min, followed by two washes with H_2_O and air-drying. Bacteria were adhered by centrifugation at 1,400 *g* for 10 min. An additional 80 µl of medium was added (180 µl total), and miniplates were loaded into a calibrated Seahorse XF HS Mini Analyzer (Agilent). KL1 (40 µM) or 0.1–0.5% DMSO was injected, and oxygen consumption rates (OCRs) and extracellular acidification rates (ECARs) were monitored at 37 °C for 4–6 h. Data were analysed using Seahorse Analytics and plotted with GraphPad Prism.

### Transcriptomic analysis

RAW 264.7 macrophages were seeded in 6-well plates (8 × 10^5^ cells per well) in 4 ml complete MEM and incubated at 37 °C and 5% CO_2_ for 16 h. Cells were infected with *S. aureus* JE2-lux at MOI 20 for 35 min, then incubated in fresh media with 100 µg ml^−1^ Gen and 40 µM KL1 or 0.1% DMSO for 24 h. Cells were washed with PBS, dissociated in 10 mM EDTA (2 ml per well) at 37 °C for 5 min, and washed three times in cold PBS. Viable cells (2.4–4.2 × 10^6^) were pelleted and submitted to Azenta for RNA sequencing (paired-end, 30 million reads per sample). Reads were aligned to the mouse GRCm38 genome using STAR v.2.5.2b.

### Databases

Biological test results of the identified compound KL1 (PubChem CID: 2881454) were retrieved from PubChem (https://pubchem.ncbi.nlm.nih.gov/)^39,76^. Gene expression profiles were examined using Expression Atlas (https://www.ebi.ac.uk/gxa/home)^77^. Biological functions and subcellular localization data were sourced from the UniProt Knowledgebase (https://www.uniprot.org/)^36^. Protein association networks among the differentially expressed genes were analysed using STRING (https://string-db.org/)^78^. Chemical structures were generated using ChemDraw software 21.0.0 (PerkinElmer).

### Reporting summary

Further information on research design is available in the Nature Portfolio Reporting Summary linked to this article.

## Supplementary information

Supplementary Information Supplementary notes, Figs. 1 and 2, Tables 1–3, legends for Table 4 and Videos 1–3.

Reporting Summary

Supplementary Table 4 SMILES of the UNC CICBDD 5 K compound library.

Supplementary Video 1 Confocal z-sections of the representative macrophages infected with an inducible GFP reporter *S. aureus* strain (green).

Supplementary Video 2 Live imaging of representative macrophages infected with an inducible mKate reporter *S. aureus* strain (red). Cells were stained with Hoechst 33342 (blue) and LysoTracker DND-26 (green) to visualize the nucleus and lysosomes.

Supplementary Video 3 Confocal z-sections of representative *S. aureus* (red)-infected macrophages. Cells were stained with Hoechst 33342 (blue) and LysoTracker DND-26 (green) to visualize the nucleus and lysosomes.

## Source data

Source Data Fig. 1 Statistical source data.

Source Data Fig. 2 Statistical source data.

Source Data Fig. 3 Statistical source data.

Source Data Fig. 4 Statistical source data.

Source Data Fig. 5 Statistical source data.

Source Data Fig. 6 Statistical source data.

Source Data Extended Data Fig. 1 Statistical source data.

Source Data Extended Data Fig. 2 Statistical source data.

Source Data Extended Data Fig. 3 Statistical source data.

Source Data Extended Data Fig. 4 Statistical source data.

Source Data Extended Data Fig. 5 Statistical source data.

Source Data Extended Data Fig. 6 Statistical source data.

Source Data Extended Data Fig. 8 Statistical source data.

Source Data Extended Data Fig. 9 Statistical source data.

## Data Availability

Bulk RNA-sequencing data generated in this work have been deposited in the NCBI’s Gene Expression Omnibus database (GEO accession number: GSE280093). Source data are provided with this paper.
