# Supplementary material for: A host-directed adjuvant sensitizes intracellular bacterial persisters to antibiotics
Source: Nat Microbiol. 2025 Oct 10;10(11):3013–25. doi: 10.1038/s41564-025-02124-2 (PMC12578635; doi:10.1038/s41564-025-02124-2)
Supplement: Supplementary file 1 — Supplementary notes, Figs. 1 and 2, Tables 1–3, legends for Table 4 and Videos 1–3. [file 41564_2025_2124_MOESM1_ESM.pdf]

nature microbiology

Supplementary information

https://doi.org/10.1038/s41564-025-02124-2

A host-directed adjuvant sensitizes intracellular bacterial persisters to antibiotics

In the format provided by the authors and unedited

Table of Contents

Supplementary notes……………………………………………..1
Supplementary figure 1…………………………………………..6
Supplementary figure 2…………………………………………..7
Supplementary table 1……………………………………………8
Supplementary table 2……………………………………………9
Supplementary table 3……………………………………………10
Legends for Supplementary table 4………………………………..11
Legends for Supplementary video 1–3……………………………11

Supplementary notes

Chemical synthesis of KL1 and KL7

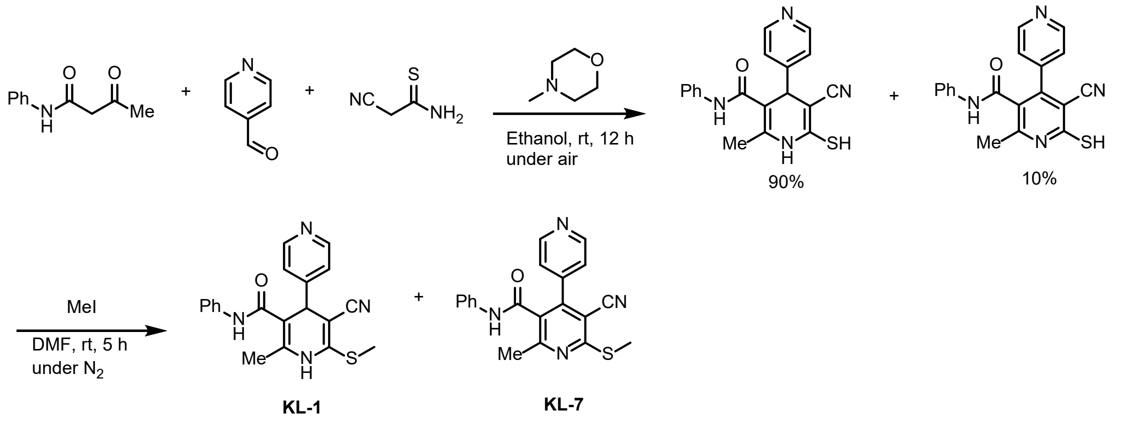

4-Methylmorpholine (3.29 mL, 30 mmol) was added dropwise to a solution of 3-oxo-N-phenylbutanamide (3.54 g, 20 mmol), isonicotinaldehyde (2.14 g, 20 mmol) and 2-cyanoethanethioamide (2.00 g, 20 mmol) in 150 mL ethanol. The reaction mixture was stirred at room temperature for 12 h under air atmosphere. Afterwards, volatiles were removed, yielding a viscous oil, which was redissolved in 20 mL dichloromethane (DCM). Then, 150 mL of hexane was added to the solution. The mixture was stirred for an additional 10 min at room temperature, resulting in a slurry. The slurry was filtered, and the solid residue was washed with a mixture of DCM/hexane (1:15, v/v). The product was dried under high vacuum to give 5-cyano-6-mercapto-2-methyl-N-phenyl- 1,4-dihydro-[4,4'-bipyridine]-3-carboxamide with >90% purity (4.02 g, 10.40 mmol).

MS (ESI): m/z calculated for $C_{19}H_{17}N_4OS$: 349.11 $[M + H]^+$; found 349.10.

100 mg of the crude product was further purified by normal-phase ISCO chromatography. The isolated impurity was isolated and characterized as the oxidized analog 5-cyano-6-

mercapto-2-methyl-N-phenyl-[4,4'-bipyridine]-3-carboxamide, as the major byproduct.

MS (ESI): m/z calculated for $C_{19}H_{15}N_4OS$: 347.10 [M + H]$^+$; found 347.10.

^{1}**H NMR** (400 MHz, DMSO-*d₆*) δ 14.52 (s, 1H), 10.33 (s, 1H), 8.71–8.65 (m, 2H), 7.48–7.42 (m, 2H), 7.34–7.20 (m, 4H), 7.10–7.01 (m, 1H), 2.49 (s, 3H).

For methylation, 538 μL methyl iodide (MeI, 8.61 mmol) dissolved in 3 mL dimethylformamide (DMF) was added dropwise to a solution of the crude 5-cyano-6-mercapto-2-methyl-N-phenyl-1,4-dihydro-[4,4'-bipyridine]-3-carboxamide (3.33 g, 8.61 mmol) in 50 mL DMF. The mixture was stirred at room temperature for 5 h under nitrogen atmosphere. Water was then added, and the mixture was extracted three times with ethyl acetate (EA). The organic phases were combined, washed with brine, dried over sodium sulfate and filtered. Volatiles were removed, and the residue was purified by normal-phase ISCO chromatography. The crude product was further slurried with a mixture of DCM/hexane (1:15, v/v), affording 5-cyano-2-methyl-6-(methylthio)-N-phenyl-1,4-dihydro-[4,4'-bipyridine]-3-carboxamide (1.98 g, 5.46 mmol) (**KL1**) with >99% purity.

MS (ESI): m/z calculated for $C_{20}H_{19}N_4OS$: 363.13 [M + H]$^+$; found 363.20.

^{1}**H NMR** (400 MHz, DMSO-d_6) δ 9.73 (s, 1H), 9.18 (s, 1H), 8.56–8.50 (m, 2H), 7.56–7.48 (m, 2H), 7.29–7.22 (m, 2H), 7.22–7.18 (m, 2H), 7.01 (tt, *J* = 7.2, 1.2 Hz, 1H), 4.75 (s, 1H), 2.52 (s, 3H), 2.13–2.08 (m, 3H).

^{13}**C NMR** (100 MHz, DMSO-d_6) δ 165.95, 152.22, 150.05, 147.04, 138.94, 137.19, 128.56,

123.33, 122.19, 119.64, 119.35, 106.12, 83.12, 42.30, 17.07, 15.63.

Fractions containing the oxidized impurity were also collected (KL7). Volatiles were removed to yield 5-cyano-2-methyl-6-(methylthio)-N-phenyl-[4,4'-bipyridine]- 3-carboxamide as a white solid in 98% purity.

MS (ESI): m/z calculated for $C_{20}H_{17}N_4OS$: 361.11 $[M + H]^+$; found 361.10.

^{1}H NMR (400 MHz, DMSO-d_6) δ 10.45 (s, 1H), 8.74–8.65 (m, 2H), 7.51–7.45 (m, 2H), 7.40–7.32 (m, 2H), 7.32–7.22 (m, 2H), 7.12–7.03 (m, 1H), 2.70 (s, 3H), 2.64 (s, 3H).

^{13}C NMR (100 MHz, DMSO-d_6) δ 163.26, 162.54, 158.59, 149.81, 148.48, 141.85, 137.87, 128.84, 128.03, 124.35, 122.98, 119.62, 114.48, 103.13, 23.04, 13.00.

^{1}H NMR Spectrum (KL1)

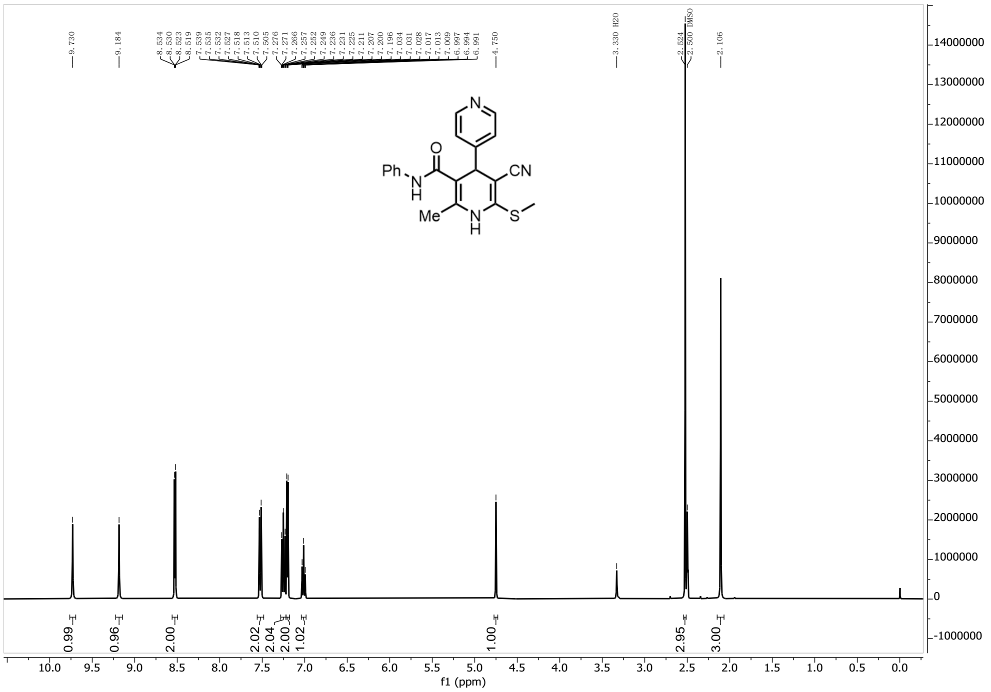

^{1}H NMR Spectrum (KL7)

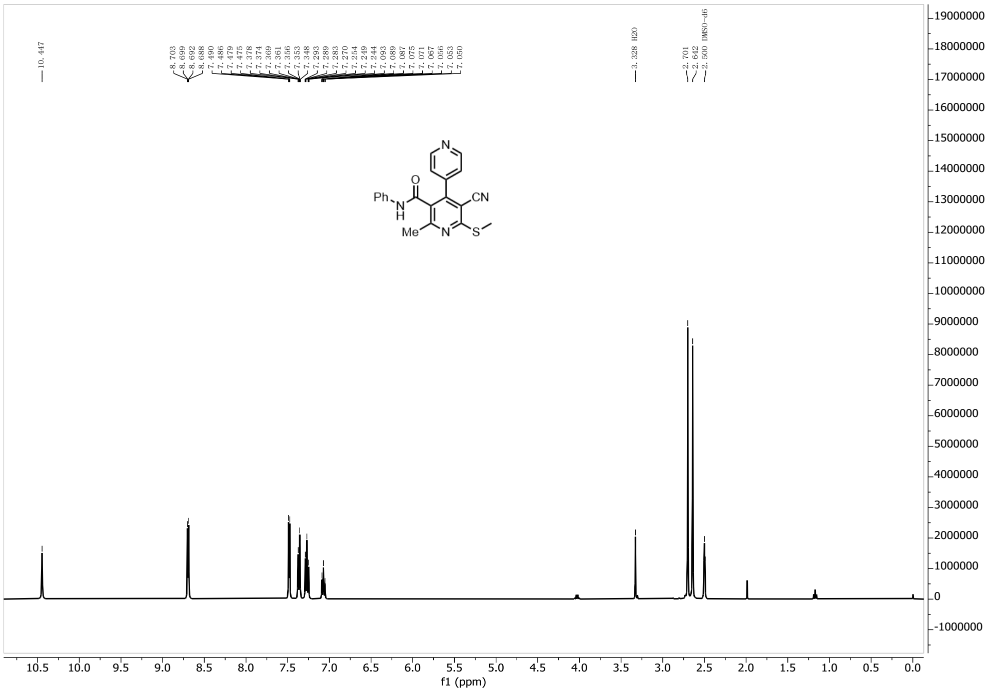

^{13}C NMR Spectrum (KL1)

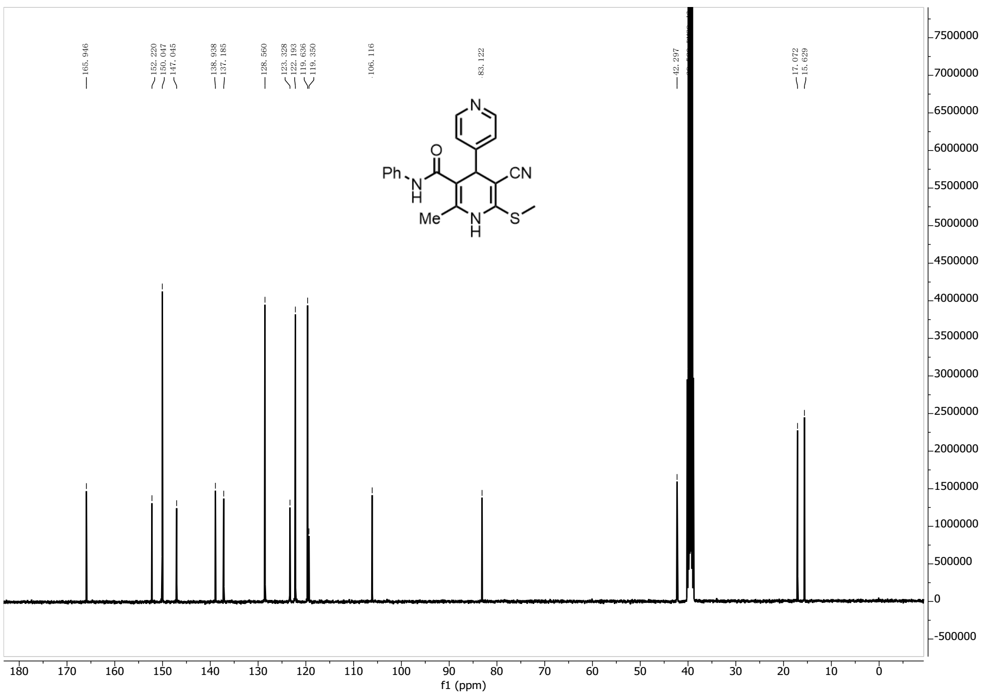

^{13}C NMR Spectrum (KL7)

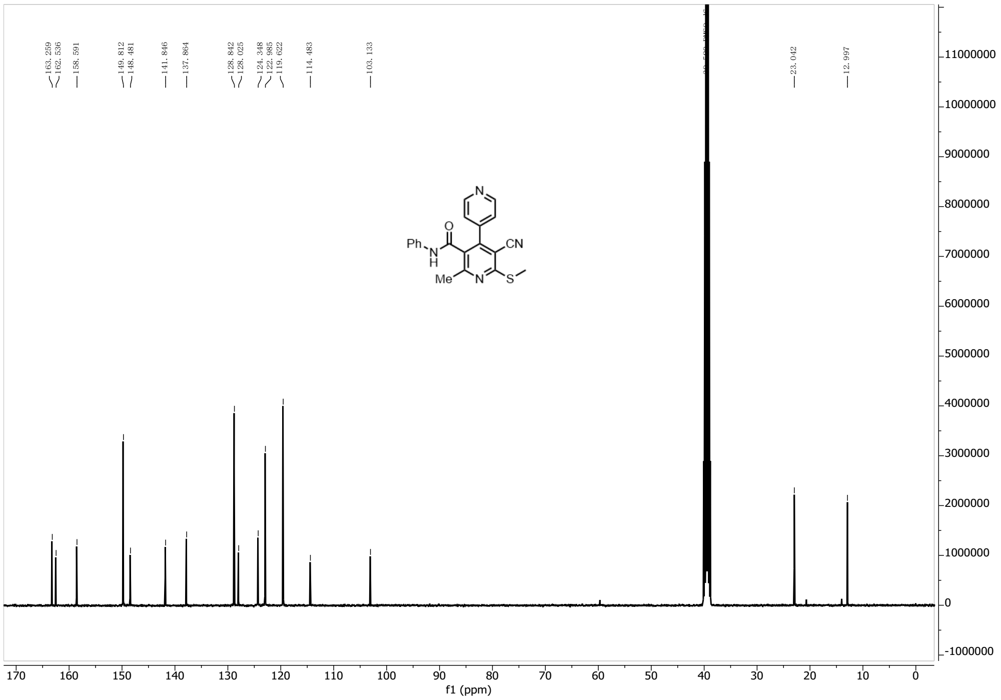

HPLC Analysis (KL1)

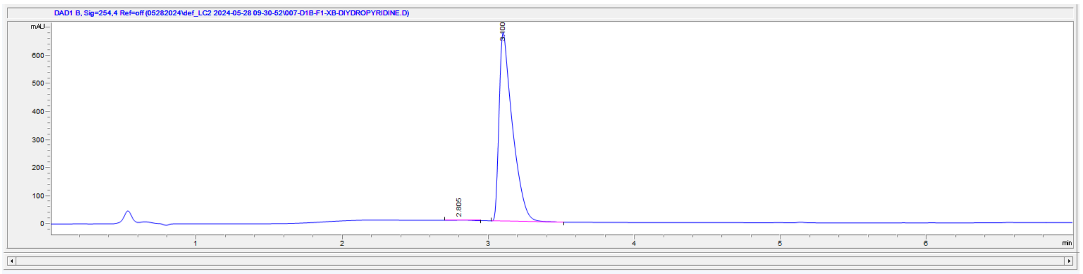

File Information

Barcode	
Operator	SYSTEM
Method	A_LCMS_DEFAULT_METHOD_POS_CH3CN.M
Reference	
Analysis Time	6.993 min
Sampling Rate	0.0067 min (0.402 sec), 1050 datapoints

#	Time	Type	Area	Height	Width	Area%	Symmetry
1	2.805	BB	10.9	1.3	0.1132	0.255	0.487
2	3.1	BB	4267	673.6	0.0919	99.745	0.404

HPLC Analysis (KL7)

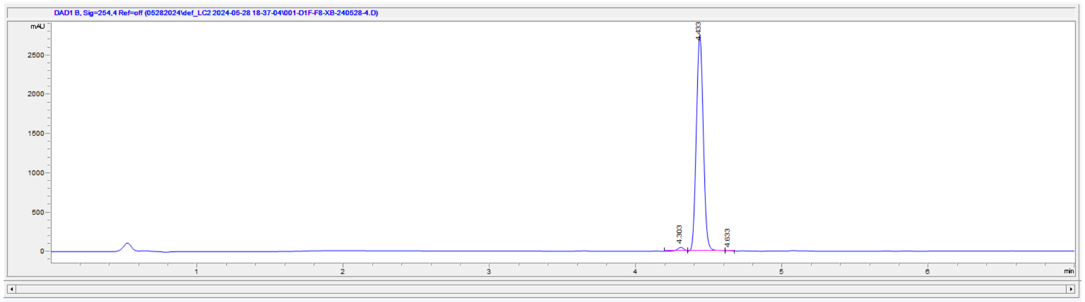

File Information

Operator	SYSTEM
Method	A_LCMS_DEFAULT_METHOD_POS_CH3CN.M
Reference	
Analysis Time	7 min
Sampling Rate	0.0067 min (0.402 sec), 1051 datapoints

#	Time	Type	Area	Height	Width	Area%	Symmetry
1	4.303	BB	141.5	46.1	0.0478	1.530	1.153
2	4.433	BB	9099.7	2766.7	0.0524	98.414	0.917
3	4.633	BB	5.1	2.3	0.036	0.056	0.396

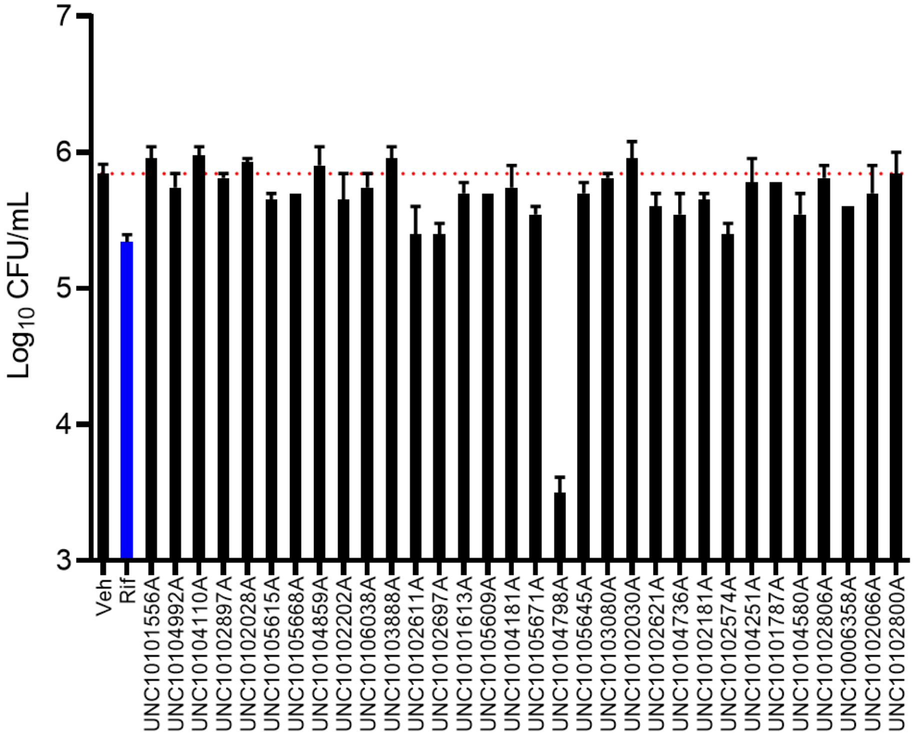

Supplementary figure 1. Compounds that most strongly reduced bacterial metabolism do not decrease intracellular persister frequencies. Of the 32 compounds that most strongly reduced bacterial metabolic activity, 31 did not reduce the burden of intracellular bacteria (n = 2). UNC10104798A appeared to lower bacterial load; however, this effect was attributable to the loss of infected macrophages during the 24-hour treatment period, rather than bactericidal activity against intracellular bacteria. Rifampicin (Rif)-treated cells were included as a reference control (blue bar). Bars represent mean ± SEM.

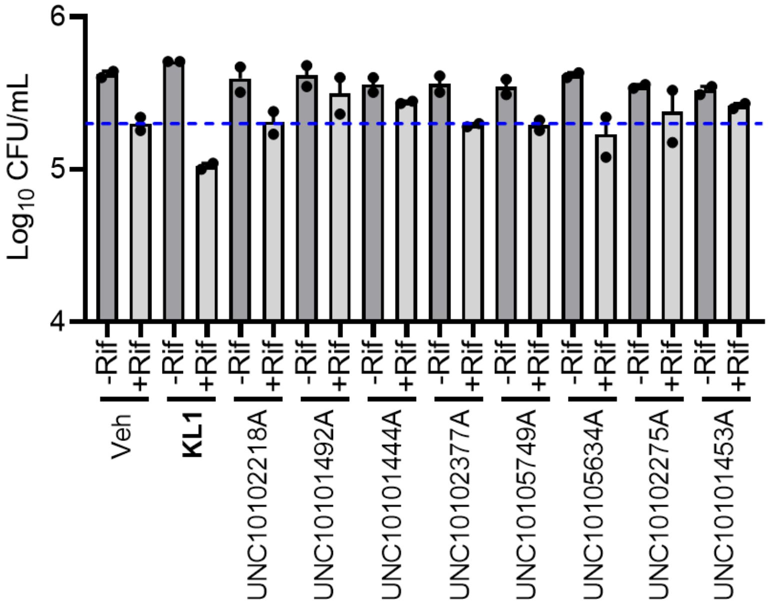

Supplementary figure 2. Only the top candidate KL1 enhances antibiotic efficacy against intracellular MRSA. Among the top nine candidate hits, only **KL1** potentiated the activity of rifampicin (Rif; 10 µg/mL) against intracellular *S. aureus* in RAW 264.7 macrophages. Gentamicin (Gen; 50 µg/mL) was included to eliminate extracellular bacteria. Results shown are from one experiment with two biological replicates (n = 2). Bars represent mean ± SEM.

Supplementary table 1. Minimum inhibitory concentrations (MICs) of antibiotics in the *S. aureus* strains used in this study.

Strains	Rif (ng/mL)	Mox (μg/mL)	Van (μg/mL)
LAC (MRSA)	8	5	1
JE2-Lux (MRSA)	6	5	1
High persister (MSSA)	6	0.16	1
Low persister (MSSA)	8	0.16	1
MW2 (MRSA)	6.25		
SA03739 (MRSA)	6.25		
SA03740 (MRSA)	6.25		
SA03758 (MRSA)	10,000		
SA03775 (MRSA)	6.25		
SA03803 (MSSA)	12.5		
SA03809 (MRSA)	6.25		
SA03815 (MRSA)	>200,000		
SA03833 (MRSA)	6.25		
SA03847 (MRSA)	>200,000		
SA03850 (MSSA)	6.25		

Three independent experiments were conducted, and the most frequently observed MIC value (i.e., the mode) was reported.

Supplementary table 2. Potential targets of KL1 based on published functional screens.

Target	BioAssay ID†	Assay	Activity	Location, expression‡
PHOSPHO1	1565	uHTS absorbance assay for the identification of compounds that inhibit PHOSPHO1	61.5% inhibition at 13.3 μM	Intracellular, immune cells and other cell types
SLC5A7	488975	Primary cell-based screen for identification of compounds that inhibit the Choline Transporter	BScore_intRatio of -5.0281	Membrane, not detected in immune cells
SLC5A7	493221	Confirmatory screen for compounds that inhibit the Choline Transporter	50.55% inhibition at 10 μM	Membrane, not detected in immune cells
EHMT2/G9a	504332	qHTS Assay for Inhibitors of Histone Lysine Methyltransferase G9a	EC_{50} value of 25.12 μM	Intracellular, immune cells and other cell types
COPS5	651999	uHTS identification of small molecule inhibitors of Csn-mediated Deneddylation of Cullin-Ring Ligases, vis a fluorescence polarization assay	93.48% inhibition at 12.5 μM	Intracellular, immune cells and other cell types

†Data sourced form PubChem biological test results.

‡Data sourced form Expression Atlas.

Supplementary table 3. Primers for molecular cloning.

Primer	Sequence
mKate forward	CAAATAGGTACCTATGTCAGAACTTATCAAGGAAAATATG†
mKate reverse	GATTACGAATTCTTAACGGTGTC
mKate_597‡	CTTGTCGGTGGAGGTCAC
mKate CTC forward	CCTTGATAAGTTCTGACATAGGTACCATCctcCTTATTTTAATTATACTCTATCAATGATAG§
mKate GAG reverse	CTATCATTGATAGAGTATAATTAAAATAAGgagGATGGTACCTATGTCAGAACTTATCAAGG§

†The sequence in red represents 5' overhang containing a KpnI site.

‡Sequencing primer to confirm the identity of the gene.

§The sequence in lowercase represent inserted nucleotides for incorporating an upstream ribosomal binding site.

Supplementary table 4. SMILES of the UNC CICBDD 5K compound library.

Supplementary video 1. Confocal z-sections of the representative macrophages infected with an inducible GFP reporter *S. aureus* strain (green).

Supplementary video 2. Live imaging of representative macrophages infected with an inducible mKate reporter *S. aureus* strain (red). Cells were stained with Hoechst 33342 (blue) and LysoTracker DND-26 (green) to visualize the nucleus and lysosomes.

Supplementary video 3. Confocal z-sections of representative *S. aureus* (red)-infected macrophages. Cells were stained with Hoechst 33342 (blue) and LysoTracker DND-26 (green) to visualize the nucleus and lysosomes.
