# Supplementary material for: A host-directed adjuvant sensitizes intracellular bacterial persisters to antibiotics
Source: Nat Microbiol. 2025 Oct 10;10(11):3013–25. doi: 10.1038/s41564-025-02124-2 (PMC12578635; doi:10.1038/s41564-025-02124-2)
Supplement: Supplementary file 2 — Reporting Summary [file 41564_2025_2124_MOESM2_ESM.pdf]

nature portfolio

Corresponding author(s): Brian P. Conlon

Last updated by author(s): Aug 1, 2025

Reporting Summary

Nature Portfolio wishes to improve the reproducibility of the work that we publish. This form provides structure for consistency and transparency in reporting. For further information on Nature Portfolio policies, see our Editorial Policies and the Editorial Policy Checklist.

Statistics

For all statistical analyses, confirm that the following items are present in the figure legend, table legend, main text, or Methods section.

n/a	Confirmed	
☐	☒	The exact sample size (*n*) for each experimental group/condition, given as a discrete number and unit of measurement
☐	☒	A statement on whether measurements were taken from distinct samples or whether the same sample was measured repeatedly
☐	☒	The statistical test(s) used AND whether they are one- or two-sided<br>*Only common tests should be described solely by name; describe more complex techniques in the Methods section.*
☒	☐	A description of all covariates tested
☐	☒	A description of any assumptions or corrections, such as tests of normality and adjustment for multiple comparisons
☐	☒	A full description of the statistical parameters including central tendency (e.g. means) or other basic estimates (e.g. regression coefficient) AND variation (e.g. standard deviation) or associated estimates of uncertainty (e.g. confidence intervals)
☐	☒	For null hypothesis testing, the test statistic (e.g. *F*, *t*, *r*) with confidence intervals, effect sizes, degrees of freedom and *P* value noted<br>*Give P values as exact values whenever suitable.*
☒	☐	For Bayesian analysis, information on the choice of priors and Markov chain Monte Carlo settings
☒	☐	For hierarchical and complex designs, identification of the appropriate level for tests and full reporting of outcomes
☐	☒	Estimates of effect sizes (e.g. Cohen's *d*, Pearson's *r*), indicating how they were calculated

Our web collection on statistics for biologists contains articles on many of the points above.

Software and code

Policy information about availability of computer code

Data collection	Confocal microscopy images and videos were captured using the Leica Microsystems and Olympus FV3000RS systems. The ImageStream images were captured using the INSPIRE software (EMD Millipore). The plate-based luminescence and fluorescence detections were performed using Synergy H1 microplate reader with Gen 5 software.
Data analysis	Statistical analyses and graph plotting were carried out using GraphPad Prism 10.3.0. For bulk RNA-sequencing, the aligned sequencing data were mapped to mouse GRCm38 reference genome available on ENSEMBL using the STAR aligner v.2.5.2b. The Seahorse data was analyzed using Seahorse Analytics (Agilent Technologies). The ImageStream images were analyzed using IDEAS 6.2 software (EMD Millipore). Confocal microscopy images were processed using ImageJ/FIJI (v1.54).

For manuscripts utilizing custom algorithms or software that are central to the research but not yet described in published literature, software must be made available to editors and reviewers. We strongly encourage code deposition in a community repository (e.g. GitHub). See the Nature Portfolio guidelines for submitting code & software for further information.

Data

Policy information about availability of data

All manuscripts must include a data availability statement. This statement should provide the following information, where applicable:

- Accession codes, unique identifiers, or web links for publicly available datasets
- A description of any restrictions on data availability
- For clinical datasets or third party data, please ensure that the statement adheres to our policy

Bulk RNA-sequencing data generated in this work have been deposited into the NCBI's Gene Expression Omnibus database (GEO accession number: GSE280093). Additional data that support the findings of this study are available from the corresponding author, Brian P. Conlon, upon request (brian_conlon@med.unc.edu).

Research involving human participants, their data, or biological material

Policy information about studies with human participants or human data. See also policy information about sex, gender (identity/presentation), and sexual orientation and race, ethnicity and racism.

Reporting on sex and gender	Sex and gender were not tracked in this study. Blood collection from healthy adult donors was used to isolate primary neutrophils and PBMCs for downstream research on antibiotic tolerance. No disease condition is under investigation.
Reporting on race, ethnicity, or other socially relevant groupings	Race and ethnicity were not tracked in this study. Blood collection from healthy adult donors was used to isolate primary neutrophils and PBMCs for downstream research on antibiotic tolerance. No disease condition is under investigation.
Population characteristics	See above.
Recruitment	All human blood samples were collected from volunteer healthy donors in the Conlon lab. All participants will provide informed consent prior to donating blood. Donors received $10/draw compensation.
Ethics oversight	The blood draw was conducted by certified personnel at the UNC Center for AIDS Research (CFAR) and performed according to standard protocols with minimal risk. The study will be conducted in compliance with ethical guidelines set forth by the institution's Institutional Review Board (IRB). The identities of the donors will be kept confidential, and all data will be handled in accordance with applicable privacy laws, including HIPAA.

Note that full information on the approval of the study protocol must also be provided in the manuscript.

Field-specific reporting

Please select the one below that is the best fit for your research. If you are not sure, read the appropriate sections before making your selection.

☒ Life sciences ☐ Behavioural & social sciences ☐ Ecological, evolutionary & environmental sciences

For a reference copy of the document with all sections, see nature.com/documents/nr-reporting-summary-flat.pdf

Life sciences study design

All studies must disclose on these points even when the disclosure is negative.

Sample size	All in vitro and tissue culture experiments were conducted using a minimum of three biological replicates. Based on previous experience with in vivo persister analysis (Conlon et al. Nature. 2013), groups of 5 mice are sufficient to achieve statistical power for assessing bacterial burdens. In this paper, we used 6 and 8 mice per group. Statistically significant changes were observed between rifampicin and KL1 co-administration and rifampicin alone groups ($p<0.001$). For Kaplan-Meier survival analysis, a total of 12 to 13 mice per group was analyzed. Improved treatment outcome was observed when co-administered with KL1 ($p<0.05$) (Mantel-Cox test).
Data exclusions	No data was excluded.
Replication	Three independent experiments with a minimum of three replicates were performed for all the in vitro and tissue culture experiments. For the animal study, two independent experiments with 6-8 male and female mice were examined.
Randomization	Mice were randomly assigned to groups.
Blinding	Blinding was not necessary as all outputs (cfu/g tissue) are objective. No subjective measurements such as clinical symptoms were measured.

Reporting for specific materials, systems and methods

We require information from authors about some types of materials, experimental systems and methods used in many studies. Here, indicate whether each material, system or method listed is relevant to your study. If you are not sure if a list item applies to your research, read the appropriate section before selecting a response.

Materials & experimental systems

n/a	Involved in the study
☒	☐ Antibodies
☐	☒ Eukaryotic cell lines
☒	☐ Palaeontology and archaeology
☐	☒ Animals and other organisms
☒	☐ Clinical data
☒	☐ Dual use research of concern
☒	☐ Plants

Methods

n/a	Involved in the study
☒	☐ ChIP-seq
☒	☐ Flow cytometry
☒	☐ MRI-based neuroimaging

Eukaryotic cell lines

Policy information about cell lines and Sex and Gender in Research

Cell line source(s)	RAW 264.7, ATCC (TIB-71);THP-1, ATCC (TIB-202); immortalized murine bone marrow-derived macrophages were generated using a CRE-J2 retroviral infection method as described.
Authentication	Cell line was not authenticated.
Mycoplasma contamination	Not tested for Mycoplasma contamination.
Commonly misidentified lines (See ICLAC register)	n/a

Animals and other research organisms

Policy information about studies involving animals; ARRIVE guidelines recommended for reporting animal research, and Sex and Gender in Research

Laboratory animals	C57BL/6J Jackson# 000664.
Wild animals	No wild animals were used in this study.
Reporting on sex	Both male and female mice were used. No sex-dependent phenotype was noted.
Field-collected samples	No field samples were collected in this study.
Ethics oversight	All animal protocols were approved by the Institutional Animal Care and Use Committee at the University of North Carolina at Chapel Hill (IACUC protocol ID: 24-029.0) and met guidelines of the US National Institutes of Health for the humane care of animals.

Note that full information on the approval of the study protocol must also be provided in the manuscript.

Plants

Seed stocks	*Report on the source of all seed stocks or other plant material used. If applicable, state the seed stock centre and catalogue number. If plant specimens were collected from the field, describe the collection location, date and sampling procedures.*
Novel plant genotypes	*Describe the methods by which all novel plant genotypes were produced. This includes those generated by transgenic approaches, gene editing, chemical/radiation-based mutagenesis and hybridization. For transgenic lines, describe the transformation method, the number of independent lines analyzed and the generation upon which experiments were performed. For gene-edited lines, describe the editor used, the endogenous sequence targeted for editing, the targeting guide RNA sequence (if applicable) and how the editor was applied.*
Authentication	*Describe any authentication procedures for each seed stock used or novel genotype generated. Describe any experiments used to assess the effect of a mutation and, where applicable, how potential secondary effects (e.g. second site T-DNA insertions, mosiacism, off-target gene editing) were examined.*
